# Longitudinal access and exposure to green-blue spaces and individual-level mental health and well-being: protocol for a longitudinal, population-wide record-linked natural experiment

**DOI:** 10.1136/bmjopen-2018-027289

**Published:** 2019-04-20

**Authors:** Amy Mizen, Jiao Song, Richard Fry, Ashley Akbari, Damon Berridge, Sarah C Parker, Rhodri Johnson, Rebecca Lovell, Ronan A Lyons, Mark Nieuwenhuijsen, Gareth Stratton, Benedict W Wheeler, James White, Mathew White, Sarah E Rodgers

**Affiliations:** 1 Swansea University Medical School, Swansea University, Swansea, UK; 2 European Centre for Environment and Human Health, University of Exeter Medical School, Knowledge Spa, Royal Cornwall Hospital, Cornwall, UK; 3 Instituto de Salud Global de Barcelona.c/ Rosselló, 132, 5º 2ª, Barcelona, Spain; 4 Research Centre in Applied Sports, Technology Exercise and Medicine, College of Engineering, Swansea University, Swansea, UK; 5 DECIPHer, Centre for Trials Research, Cardiff University, Cardiff, UK; 6 Department of Public Health and Policy, University of Liverpool, Liverpool, UK

**Keywords:** wellbeing, environmental exposure, geographic information systems, routine linked data, longitudinal, mental health

## Abstract

**Introduction:**

Studies suggest that access and exposure to green-blue spaces (GBS) have beneficial impacts on mental health. However, the evidence base is limited with respect to longitudinal studies. The main aim of this longitudinal, population-wide, record-linked natural experiment, is to model the daily lived experience by linking GBS accessibility indices, residential GBS exposure and health data; to enable quantification of the impact of GBS on well-being and common mental health disorders, for a national population.

**Methods and analysis:**

This research will estimate the impact of neighbourhood GBS access, GBS exposure and visits to GBS on the risk of common mental health conditions and the opportunity for promoting subjective well-being (SWB); both key priorities for public health. We will use a Geographic Information System (GIS) to create quarterly household GBS accessibility indices and GBS exposure using digital map and satellite data for 1.4 million homes in Wales, UK (2008–2018). We will link the GBS accessibility indices and GBS exposures to individual-level mental health outcomes for 1.7 million people with general practitioner (GP) data and data from the National Survey for Wales (n=~12 000) on well-being in the Secure Anonymised Information Linkage (SAIL) Databank. We will examine if these associations are modified by multiple sociophysical variables, migration and socioeconomic disadvantage. Subgroup analyses will examine associations by different types of GBS. This longitudinal study will be augmented by cross-sectional research using survey data on self-reported visits to GBS and SWB.

**Ethics and dissemination:**

All data will be anonymised and linked within the privacy protecting SAIL Databank. We will be using anonymised data and therefore we are exempt from National Research Ethics Committee (NREC). An Information Governance Review Panel (IGRP) application (Project ID: 0562) to link these data has been approved.

The research programme will be undertaken in close collaboration with public/patient involvement groups. A multistrategy programme of dissemination is planned with the academic community, policy-makers, practitioners and the public.

**Strengths and limitations of this study**

- This retrospective controlled evaluation of a natural experiment includes the majority of adults in Wales, UK between 2008 and 2018, minimising selection bias.
- Generating a national longitudinal dataset of changes in green-blue spaces (GBS) exposure and access to GBS for households will reduce ecological fallacy.
- Spatial and temporal accessibility data linked for individuals and their routinely captured health service use, together with potential confounders, will allow us to investigate the impact of GBS on well-being.
- Detailed self-report data on well-being and GBS visit behaviour from cross-sectional national survey data will be linked to health service utilisation data.
- Despite including a large number of potentially confounding variables, this non-randomised study using routinely recorded data may omit some unknown confounders, thereby introducing a moderate level of bias due to confounding.

## Introduction

Globally, 686 million people suffer with common mental health disorders (CMDs) such as depression or anxiety.^1^ In the UK, CMDs are experienced by around one in four of the population, and mental ill health costs the economy over £100 billion per annum in health, social care and quality of life loss costs.^2 3^ Subjective well-being (SWB) is also related to mental and physical health outcomes, including life expectancy,^4 5^ and is a key marker of quality of life.^6^ With increasing impacts on wider societal costs, CMDs and promoting SWB are growing in importance. Access to natural environments—considered here as ‘green-blue spaces’ (GBS)—such as parks and beaches—may provide opportunities to support and promote good public mental health and well-being.^7 8^ The evidence base on the impacts of GBS, on mental health and well-being is growing rapidly. Current research suggests that the benefits may differ by population group, context and health outcome.^9 10^

Recent systematic reviews, of predominantly cross-sectional studies, indicate positive relationships between mental health and SWB outcomes with living near GBS.^11 12^ Access to GBS is positively associated with GBS use, and using GBS may improve health outcomes through a number of mechanisms. For example, increased physical activity,^13 14^ psychological restoration,^15 16^ noise mitigation,^17^ heat and humidity regulation,^18 19^ increased social interaction and cohesion.^20–22^ Contrary to these findings, a study in New Zealand found no evidence that GBS influenced cardiovascular disease mortality^23^ and suggested that GBS and health relationships may vary according to country or environmental contexts.

Many studies have found that access to GBS can vary across socioeconomic status (SES) areas; with more deprived areas tending to have poorer access to GBS.^24 25^ Differences in the distribution of GBS across SES may influence and contribute to SES-related health inequalities.^26^ Furthermore, individuals from higher SES groups are more likely to select living in greener neighbourhoods.^27^ Previous studies lack censoring for births, deaths and migration, which may have led to health selection effects which can alter the strength of association. There is also a lack of large-scale population-level studies which have systematically and explicitly examined associations within and between subgroups other than SES^10^ (eg, by gender,^28–30^ age, education^22 31^). Furthermore, ethnic minority groups are under-represented in studies due to selective non-response.^32^ Studies that have focused on differences between ethnic minorities found positive effects of GBS on well-being differed by ethnicity^33^ and ethnic minorities had poorer access to GBS.^34^

Study methods used to define access to GBS differ; some studies have calculated small area-level metrics and others have used distances. There is no accepted method to define GBS or for measuring different types of GBS (eg, according to general land cover categories, ecosystems or landscape classifications).^35–41^ However, it is difficult to expect an authoritative voice on defining GBS access because how people engage with GBS varies by population subgroup and environmental context. Therefore, how GBS is defined will vary by study design and the focus of the research. We propose to take an approach that will consider land use but also access points, rights of way and amenities such as benches. There are also differences in study aims because of differing perspectives; such as health research, environmental research and policy. Few studies have also considered the issue of access to blue spaces,^42 43^ which has also contributed to diversity in GBS accessibility measures. Although there is now a significant body of research, there are few longitudinal studies on mental health outcomes in response to changes in GBS access or with assessments of actual visits to GBS.^44–47^

**Figure 1:**
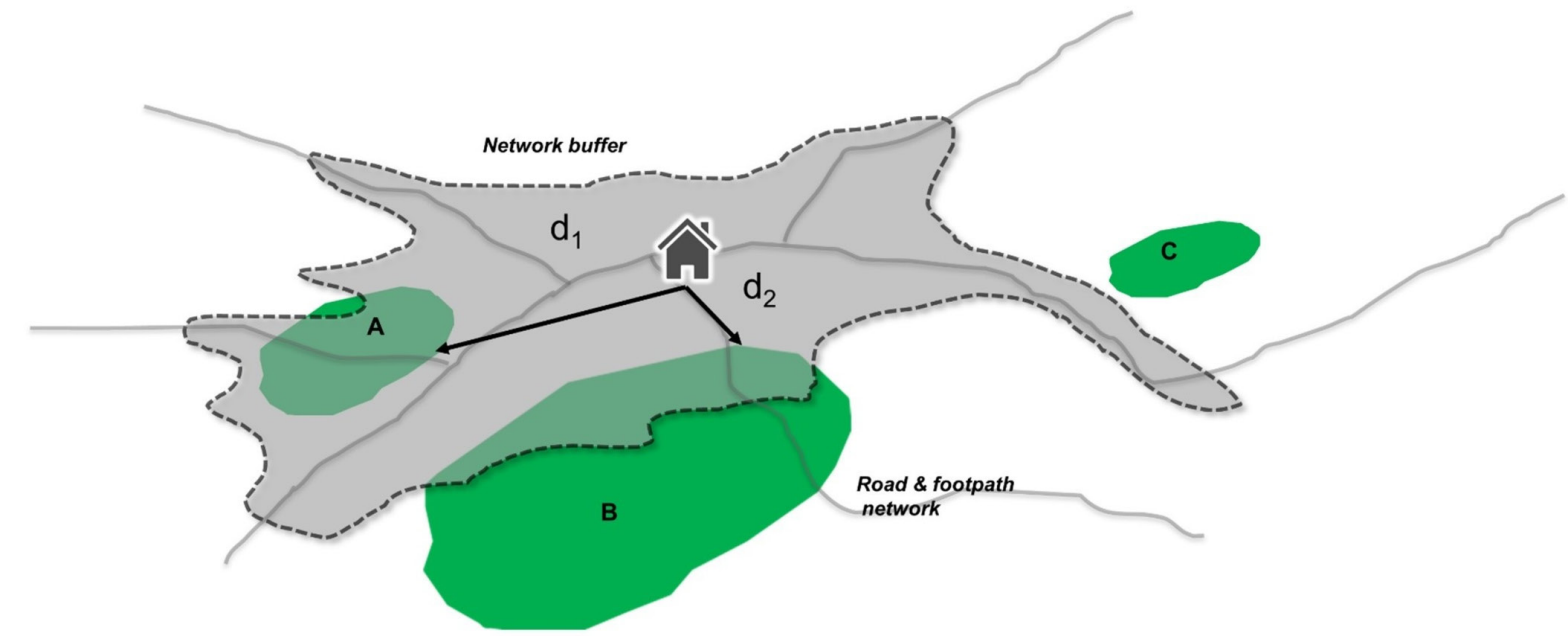
Residential GBS access for each home. (1) Network buffer defines household access boundary (eg, 10 min walk). (2) GBS A and B included in access estimates including access points, rights of way and facilities such as benches (GBS C excluded). (3) Access measures include GBS quality, size, function and weighted network distances (d_i_) to access points. (4) Longitudinal exposure estimates created through quarterly repeat analysis. (5) GBS, green-blue spaces.

We define GBS accessibility indices as metrics that represent how people may access GBS (figure 1). GBS exposure is the ambient environment that people experience because of where they live and will be defined as greenness immediately surrounding home/can be seen out of the window. By including both of these measures in our study, we will be able to build a more holistic view of how GBS impacts CMD and well-being.

### Aim

Our primary aim is to quantify the impact of changes in access to GBS and ambient GBS exposure on CMD and SWB, for a national population. This will be fulfilled through a number of objectives within two work packages (WPs). WP1 will use routinely collected health and demographic data (data collected for purposes other than research^48^) stored in an anonymised databank to examine the risk of CMD using longitudinal changes in access to neighbourhood GBS. WP2 will link routine data in the anonymised databank with in-depth survey responses from the data linked National Survey for Wales (NSW) to evaluate a representative sample of Welsh residents’ self-reported well-being and GBS use.

### Objectives

#### WP1

1. Create a longitudinal dataset of GBS in Wales using UK Ordnance Survey (OS), local authority and remotely sensed satellite data.
2. Create longitudinal, residential GBS accessibility indices for all homes in Wales using the longitudinal GBS dataset in a Geographic Information System (GIS).
3. Create an 11-year dynamic cohort of individual-level longitudinal residential GBS accessibility indices to answer the research question(s): ‘Do people with different GBS access through time have different associated risks of having a CMD?’ and ‘Is the association between changes in access and exposure to GBS and CMD modified by multiple sociophysical variables, migration and socioeconomic disadvantage?’

### WP2

1. Create data linkages between survey and routinely collected data within the Secure Anonymised Information Linkage (SAIL) for household-level GBS accessibility indices, and individual-level health and demographic data.
2. Complement residential GBS accessibility indices by including GBS usage from the data-linked NSW to model interactions to answer research question: ‘Are there associations between residential GBS access and exposure, and well-being and CMDs? Are these associations modified by GBS use and multiple sociophysical modifiers?’We will consider stratifying by CMD to check for reverse causation.

## Methods and analysis

### Design

The GBS project is a retrospective and controlled, population-wide study. We will evaluate the association between changes in access and exposure to GBS, on the risk of CMD (WP1) and SWB (WP2). This could be either at environment level (eg, a change in the GBS itself) or person level (eg, moving home, better access to transport). WP1 will use longitudinal data to examine variability in time and space of access and exposure to GBS and examine whether this could be due to planning and environmental policies. WP2 will use cross-sectional data to investigate whether visits to GBS improve SWB.

### Participants

**Figure 2:**
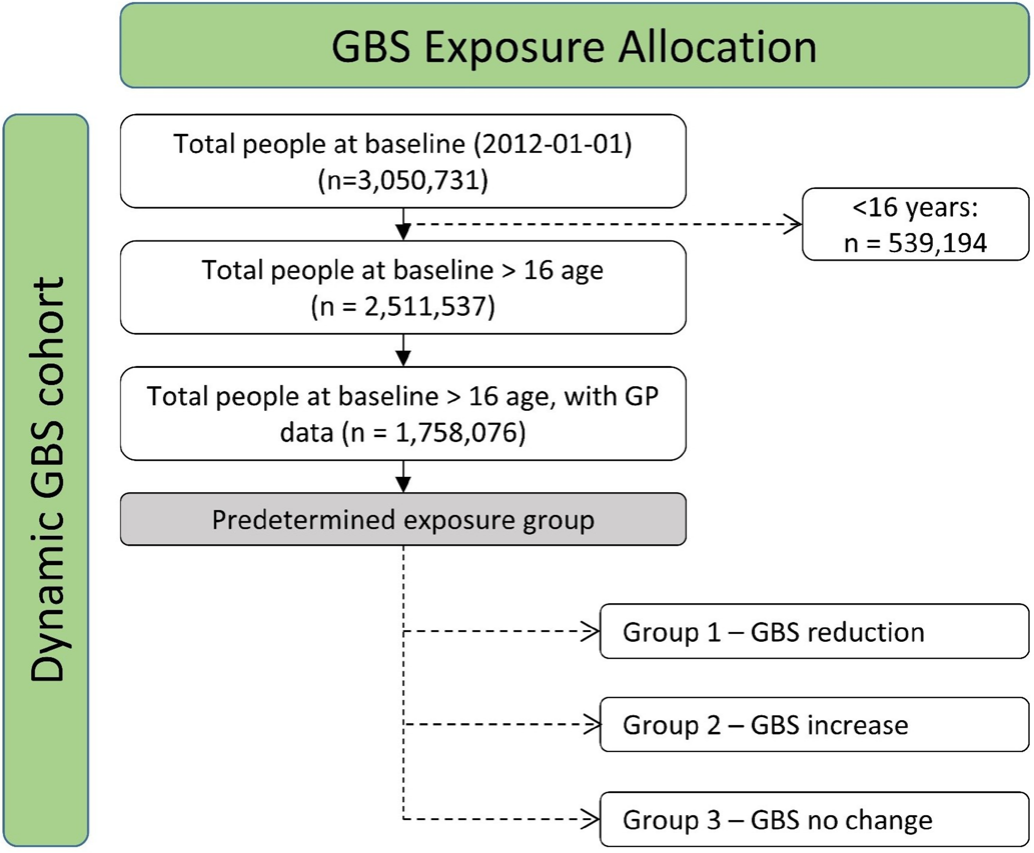
Flow diagram of proposed number (1.7 million adults) we currently have before exposure group allocation. GBS, green-blue spaces; GP, general practitioner.

Our study population contains people aged 16 years and older living in Wales, UK. WP1 includes the total adult population registered with a general practitioner (GP), providing GP records to SAIL. This is expected to be about 1.7 million adults in Wales (see figure 2). WP2 includes a representative sample of the adult population in Wales based on the NSW for 2 years (cross-sectional samples in 2016/2017 and 2017/2018). The NSW has an annual sample of approximately 12 000 responders and GBS visit questions are asked of 50% of that sample. This provides a total cross-sectional sample of 12 000 (over the 2 years) for this part of the study.

### Research environment

We will use a secure GIS platform hosted by United Kingdom Secure e-Research Platform (UKSeRP).^49^ High-resolution map data from the OS are stored in the GIS, including point data for all residences (AddressBase Premium), and road network data.^50 51^ Environment data will be collected, stored and processed to generate household - level GBS accessibility indices.

In parallel with, but separate from the GIS, is the SAIL databank.^52–54^ Data linkage completed at our Trusted Third Party, will enable a longitudinal cohort to be created before statistical analysis is conducted within SAIL. The SAIL Databank contains longitudinal health, social and education data formore than 5 million people, which includes the current population of Wales, UK (3.1 million people) at any one time. The databank includes over 15 billion anonymised records and was designed to overcome data sharing issues.

**Figure 3:**
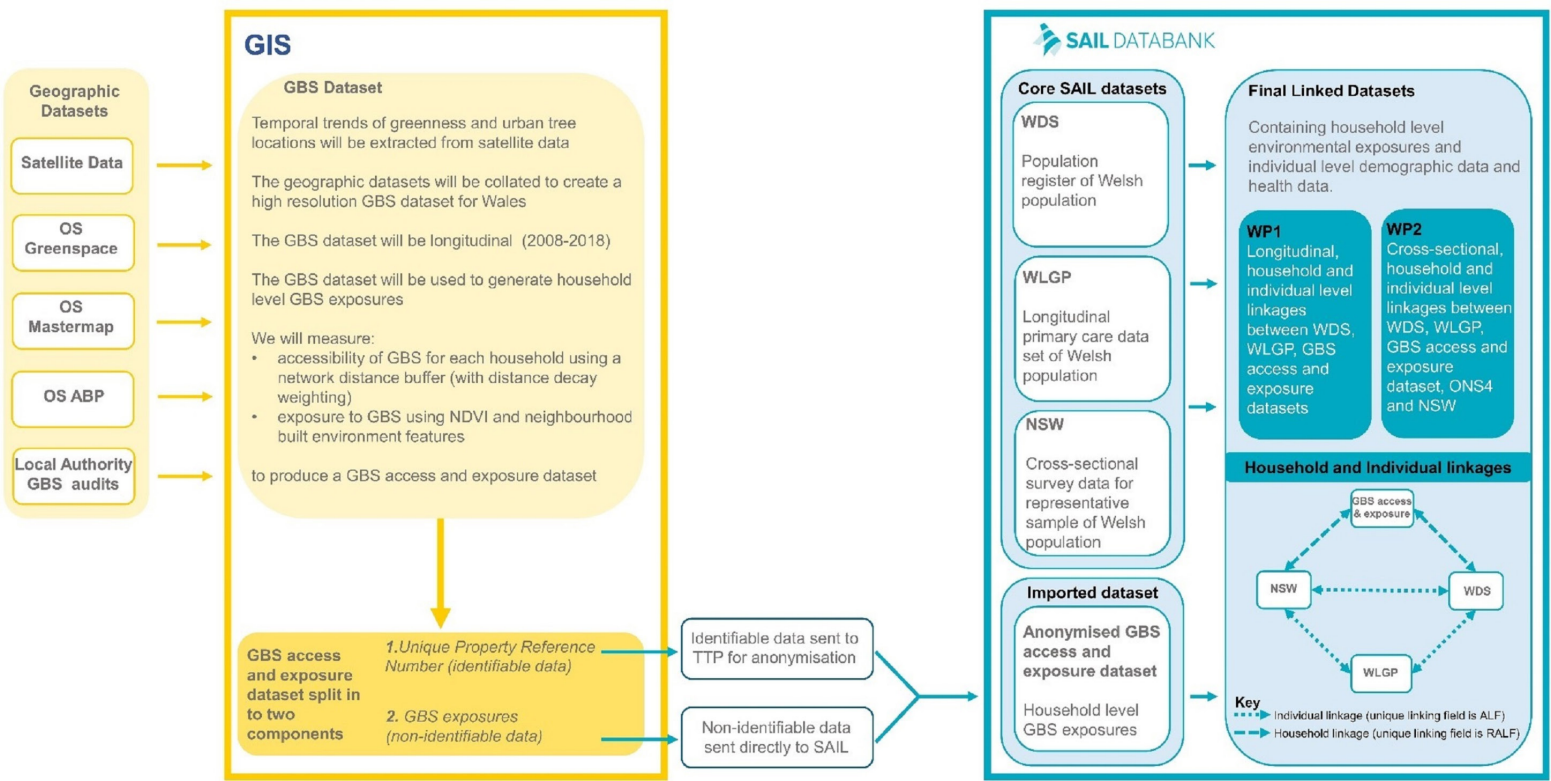
Diagram of proposed GIS data preparation and data linkage in SAIL databank. GBS accessibility and exposure indices created within a secure GIS platform will be linked at household level with demographics from Welsh Demographic Service (WDS) and National Survey for Wales (NSW). Common mental health conditions from GP records will be linked at individual level with the NSW and WDS. Linkage will be undertaken by our TTP (NHS Wales Informatics Service). ABP, AddressBase Premium; ALF, Anonymised Linking Field; GBS, green-blue spaces; GP, general practitioner; GIS, Geographic Information System; NDVI, Normalised Difference Vegetation Index; ONS, Office for National Statistics; OS, Ordnance Survey; RALF, Residential Anonymised Linking Field; SAIL, Secure Anonymised Information Linkage; TTP, Trusted Third Party; WDS, Welsh Demographic Service; WLGP, Welsh Longitudinal General Practice; WP1, work package.

A strength of the SAIL platform is the method for anonymising all individuals and households in Wales. Data are anonymised through the split file process.^52 53^ Whereby the dataset is separated into identifiable (eg, address) and non-identifiable (GBS access and exposure) components. The identifiable component is transported to our trusted third party (TTP), NHS Wales Informatics Service.^55^ The non-identifiable component is sent securely to the SAIL Databank. The TTP anonymise and encrypt the identifiable data and each individual record is assigned a unique linking field. An Anonymised Linking Field (ALF) is assigned to individuallevel data and a Residential Anonymised Linking Field is assigned to a place of residence.^55^ The anonymised elements of the dataset are then sent to SAIL to be loaded. These elements are then recombined with the non-identifiable (GBS access and exposure) component of the dataset, which makes them ready for linkage with other datasets in the SAIL Databank (see figure 3).

### Data sources

#### Environment datasets

We will create a longitudinal, national dataset of GBS for Wales (2008–2018) using temporally and spatially referenced satellite imagery.^56^ We will also develop a typology of GBS and collate data from multiple sources to augment the all-Wales OS data we hold in our GIS database (OS Mastermap).^50 51 57^ Using satellite imagery, we will extract temporal trends of ‘greenness’ (using measures such as the Normalised Difference Vegetation Index (NDVI), which has been used in previous studies^58^) for every household in Wales and we will also explore the integration of new GBS and land use data as they become available and are published (eg, OS Greenspace data^59^) and local authority audits of GBS.

### GBS accessibility and exposure dataset

There is no clear consensus in the existing literature on the most appropriate measures of GBS access and/or exposure.^42^ Using the aforementioned environment datasets, this project will collate a set of measures using criteria including longitudinal consistency, spatial resolution and evidence for associations with mental health and SWB outcomes. We will create residential-level exposure measures by modelling the ambient home environment, that is, the GBS immediately surrounding their home and what they see out of their window. This metric will include measures such as greenness (NDVI), urban trees and garden size. The access measures will represent people’s potential to visit GBS in their locality. We will adapt a previous methodology that modelled ‘change in alcohol outlet density and alcohol-related harm to population health’ (CHALICE).^60^ The distance decay for this accessibility model will be updated to emulate how people engage with GBS as opposed to alcohol outlets. People will behave differently in accessing GBS, compared with how they may access alcohol outlets. For example, people may be prepared to walk or drive further to access some green blue spaces compared with alcohol outlets.^61^ Our modelling will consider differential associations for key subgroups (eg, deprived populations) thereby minimising risks that recommendations could increase inequalities. We will create a number of accessibility and exposure measures, allowing planners to consider the configuration (size, function, quality) of the most beneficial mix of spaces. We will also measure, for each adult, change in GBS access and exposure for the study period. Again, we will build on the CHALICE study^62^ that measured change in alcohol outlet density by calculating change in density between the current and previous quarter and also the change between each quarter and baseline.

The longitudinal, household-level, GBS accessibility and exposure dataset will be imported into the SAIL databank and linked with health datasets that are held within the SAIL Databank. Household identifiers (Unique Property Reference Numbers, UPRNs) will be used to distinguish each household in the GIS and will have attached to each a GBS accessibility and exposure index (see figure 3). Household identifiers will be replaced with a Residential Anonymised Linking Field within SAIL.^63^

### Health datasets

#### Welsh Longitudinal General Practice

The Welsh Longitudinal General Practice (WLGP) dataset contains individual-level health data including Read codes^64^ for all diagnoses, symptoms and treatments recorded by a GP and we will extract outcome data for each person from 2008 to 2018.

### Welsh Demographic Service

The Welsh Demographic Service (WDS) dataset contains addresses for all individuals who register with a GP. Dates for each address record update are recorded, thereby providing durations of residency for multiple homes and the ability to link to local environment indices at each time point. This dataset holds demographic data including age and gender. These data will be used to create population subgroups based on age, gender and location for each period. The WDS contains historical patient provided address information linked anonymously at the individual level (the ALF) which is the primary key variable for record linkage.^52 53^

### National Survey for Wales

The NSW is a cross-sectional, representative sample of adult participants across Wales. Participants answer questions on public services such as education, transport, leisure activities, and self-reported health and well-being. They also report information on visits to GBS, such as visit frequency and activities undertaken during visits.

Figure 3 shows how GBS accessibility indices will be generated and linked with routinely collected health and survey data held within the SAIL Databank. Table 1 shows the data sources and variables that we plan to derive from the routinely collected health data, survey dataset, and population register that are available in the SAIL Databank.

**Table 1:** Data sources for outcome and accessibility/exposure variables to be used in statistical analysis for years 2008–2018

Dataset name	Data source	Derived variables	Coverage	Work package (WP)
GBS accessibility and exposure dataset.	Local authorities, ordnance survey, satellite data sources.	Typology to be developed. The dataset will include GBS such as parks, woodland, sports facilities, trees in residential areas.	Wales.	WP1 and WP2
Welsh Longitudinal General Practice dataset (core SAIL dataset).	Primary care records recorded by GP.	CMDs such as anxiety and depression (see outcome variable section for details).	70% of practices in Wales provide data to SAIL.	WP1 and WP2
Welsh Demographic Service dataset (core SAIL dataset).	NHS Wales Informatics Service.	Age, gender, week of birth, multiple move in and out of home dates.	Total population of Wales who are registered with a GP, a free to use service in the UK.	WP1 and WP2
National Survey for Wales (restricted SAIL dataset).	Welsh Government.	Subjective well-being, self-reported visits to GBS.	Cross-sectional, representative sample approximately 12 000 participants per year.	WP2

GBS, green-blue spaces; GP, general practitioner; CMDs, common mental health disorders; SAIL, Secure Anonymised Information Linkage.

## Statistical analysis

### Outcome variables

#### WP1 and 2: primary outcome - CMD

**Figure 4:**
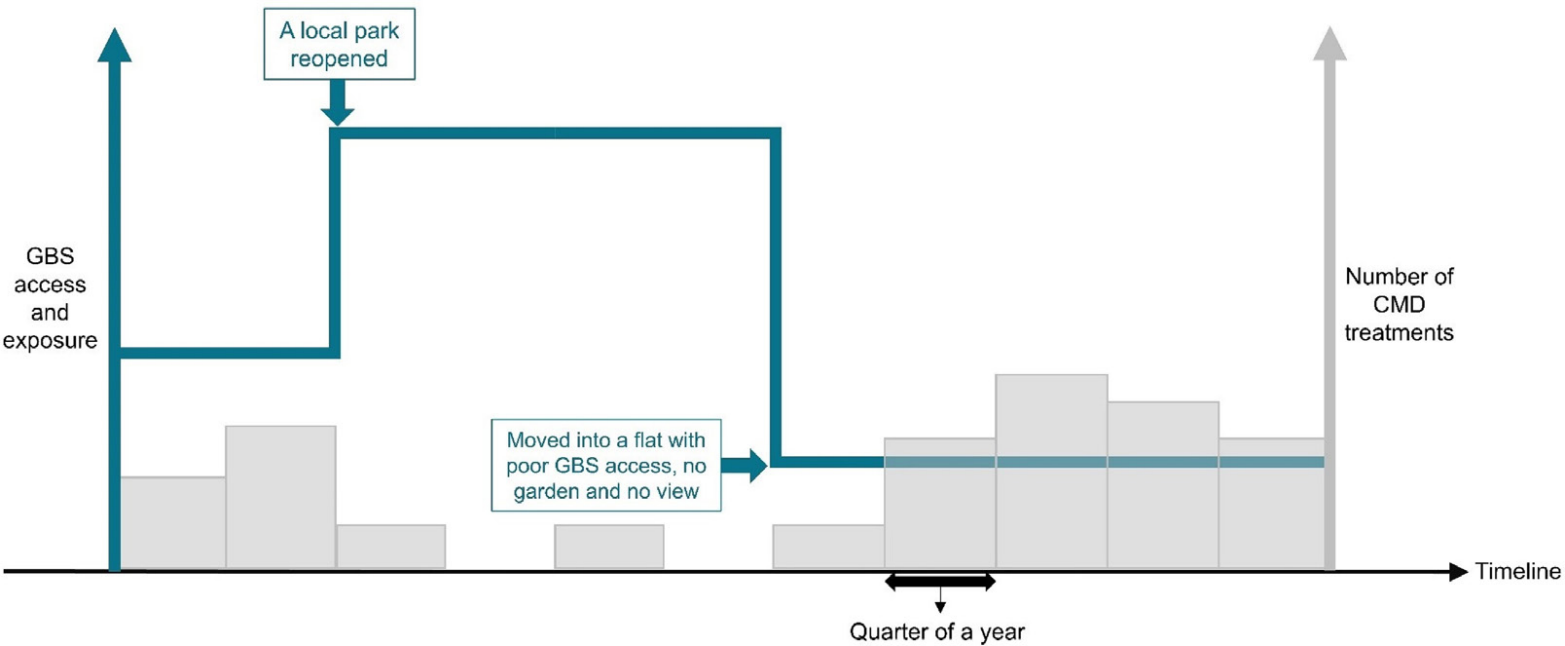
Conceptual model of primary exposure variable and primary outcome variable. CMD, common mental health disorder; GBS, green-blue spaces.

Change in counts of CMD treatments for adults identified as CMD cases within the corresponding time periods for the 70% of adults in Wales for whom we have GP data records in SAIL (1.7M adults). Prevalence algorithms,^65 66^ detect cases of CMD (anxiety and depression) from routinely collected GP data. We will use the algorithm that incorporates: an historical diagnosis and currently treated, with current diagnosis whether treated or untreated, to identify CMD cases. For each adult, and for the time periods (quarters) identified as a CMD case, treatments will be counted per day and aggregated into quarterly counts. Figure 4 shows a conceptual model of exposure variable (GBS access and exposure) and primary outcome variable (CMD treatments).

#### WP2: secondary outcome - SWB

SWB is measured by the Warwick-Edinburgh Mental Well-being Scale (WEMWBS) in the NSW for two survey years (2016/2017 and 2017/2018) for a representative population.^67^ The WEMWBS is comprehensive (incorporating elements of both SWB and psychological well-being), short enough to be used in population-level surveys, responsive to change and has been validated among community samples of adults in the UK.^68^ WEMWBS scores are on continuous scale from 14 to 70.

#### WP1: secondary outcome - GP events

We have the total number of events recorded for each person in a WLGP dataset. We will calculate the number of GP events, converting to a binary daily activity or no activity aggregated to quarterly counts. This eliminates double counting (eg, counting the return of large quantities of test results only once). These are purposefully all events rather than those related only to CMD because this will capture people who frequently visit their GP but for whom no CMD diagnosis has been made. This will allow us to explore social isolation and as indicator of undiagnosed CMD.

#### WP2: secondary outcome - Office for National Statistics SWB

We will have individual responses in NSW to the four standard SWB questions (life satisfaction, sense of worth of activities, happiness, and anxiety) as used by Office for National Statistics and the Organisation for Economic Co-operation and Development.^69^ As well as being relatable to national representative survey data, these data have also been used in previous GBS visit research using the comparable Monitor of Engagement with the Natural Environment for England.^45^

### Analysis plan

We will provide descriptive statistics of GBS access and exposure and changes in GBS access and exposure by household, Lower Layer Super Output Area (LSOA), deprivation quintiles using Welsh Index of Multiple Deprivation (WIMD) and population characteristics. NSW data will have descriptive statistics of each of the survey questions we will use (number of visits, labour market status, etc).

Across both WPs, we have outcomes of two distinct types: continuous (SWB) and counts (CMD treatments and GP event days). We intend to analyse the continuous outcome using a linear model. Poisson models will be used to analyse the longitudinal count data.

Our data are hierarchical in nature: WP1: three-level data: LSOA/Individual/Time (Quarter); WP2: two-level data: LSOA/Individual. Accordingly, we will adopt a multilevel modelling approach. We will generalise the standard linear and Poisson models to handle two or three levels of variation, as appropriate. The resulting two-level and three-level models will allow us to estimate the extent to which variation at each level may be explained by confounding variables (see table 2). Having controlled for confounders, we will proceed to add access and exposure variables to our models, thereby allowing us to test for the significance of the access and exposure variables in the presence of the confounders. Inclusion of these variables in the above models will permit us to estimate their direct effects on our outcomes. Having included the main effects of both confounders and access and exposure variables, we will proceed to test for the significance of selected pairwise interactions of interest, as shown in table 2.

**Table 2:** Analysis summary (P: primary; S: secondary)

WP	Outcomes (type)	Exposures	Confounders	Interactions of interest
1	P: CMD (count)	P: Access and exposure to GBS.	LSOA level: Quintile of deprivation (WIMD). Category of urban/rural settlement type (ONS classification).	Change in access and exposure to GBS by deprivation.
	S: GP event days (count)	S: Time of move(s).	Individual level (SAIL): gender, age, comorbidities.	Change in access and exposure to GBS by time of move(s).
2	P2: SWB (continuous)	P2: Access and exposure to GBS.	LSOA level: as per WP1.	Exact number of visits by individual level deprivation.
	P1: CMD (count)	S: Level of engagement in 150+ min of moderate or vigorous intensity activity per week.	Individual level and NSW: gender, age, comorbidities, highest educational qualification, marital status (incl. living with partner).	Exact number of visits by residential GBS.
			Exact number of visits made outdoors for recreation in last 4 weeks.	

CMD, common mental health disorder; GBS, green-blue spaces; GP, general practitioner; NSW, National Survey for Wales; ONS, Office for National Statistics; SAIL, Secure Anonymised Information Linkage; SWB, subjective well-being; WP1, work package 1.

## Discussion

This study will be the first of its kind to link GIS-generated GBS accessibility and exposure measures with routinely collected, longitudinal health data and cross-sectional survey data for a whole population. We plan to create a longitudinal GBS dataset for Wales. Using multisource data, we will build a dataset that records local-level changes in GBS for Wales, for 11 years. Longitudinal studies that have previously examined GBS exposure have used cross-sectional environment data to calculate GBS accessibility and exposure indices. This may be because it is a resource and time-intensive task to create a longitudinal GBS dataset, and longitudinal data are not always available. In addition, bringing together data from different sources, harmonising the data and deriving GBS indices from the data requires expertise and specialised skills. A national study using routinely collected data on a national scale is timely following recommendations from a report on a prospective quasi-experimental study.^47^

Our study will work with stakeholders and policy-makers to develop a GBS typology that can be used to provide evidence that can be translated to help policy and practice. Improved evidence on the impacts of GBS on mental health is required to inform decisions relating to planning, area regeneration and environmental management. Natural England, England’s statutory body responsible for the natural environment, concluded that a knowledge of causal pathways and contributory mechanisms that link mental health and environmental exposure is required.^70^ This study will produce the evidence needed to address gaps stated by the Welsh Government Environment Bill White Paper^71^ and inform implementation of the Well-being of Future Generations (Wales) Act 2015.^72^ We have key policy-makers signed up as stakeholders for this project and they will be involved in discussions for how we define GBS, how we measure access and exposure to GBS and in the reporting of our results so that this study can be useful in generating evidence-based policy.

Another aim of the study is to create novel GBS accessibility indices to use network routes from a variety of sources, and access points, to model how people may access GBS. The way that studies measure access to GBS is methodologically diverse and there is no general consensus on which is the best measure to use. Our measures will be informed by theories and findings in the literature. To complement this, we will ensure we involve members of the public in our research. The overarching aim of our longitudinal study is to identify causal mechanisms to determine whether a positive change in access and exposure to GBS lowers the risk of CMD.

### Patient and public involvement

During the development of the study exposure measurement, we engaged with the SAIL Databank consumer panel, who provided us with public perspectives. We will invite members of the public to workshops and ask them to help direct our research through a series of focused questions. The workshop group will comprise members who are experienced at considering the value of environment from the Health and Environment Public Engagement (HEPE) Group (hosted by University of Exeter), who will join Wales-based members of the public from urban park groups, and those experienced in considering data linkage proposals.^73^ All members will be updated with project progress.

### Ethics and dissemination

#### Ethical arrangements

This study is based on routinely collected administrative, environment and survey data. All data will be anonymised into a secure databank, and therefore, there will be no mechanism for informing potential study participants of possible benefits and known risks. We have obtained informed consent to use the linked and anonymised NSW data within the SAIL databank. All routinely collected anonymised data held in SAIL are exempt from consent due to the anonymised nature of the databank (under section 251, National Research Ethics Committee (NREC)).

### Research governance

We have applied and been granted approval by the independent Information Governance Review Panel (IGRP) for permission to conduct this study (study number 0562). The IGRP contains independent members from NREC and British Medical Association (BMA), as well as lay members, and have previously given permission for similar projects (eg, NIHR PHR CHALICE and NIHR PHR Carmarthenshire Housing). The review process has checked that the study we are is useful, not service evaluation, and will not break anonymisation standards.

### Dissemination policy

We will regularly report our progress to the study steering committee (SSC). The SSC will comprise academic experts and stakeholders (NRW, Keep Wales Tidy, Sport Wales and Welsh Government). At the end of the study, we will hold a workshop to report our findings to stakeholders and the public. We will disseminate our findings to patient, policy and academic networks (eg, Health Data Research UK, Administrative Data Research and National Institute for Health Research) and we will present results to the public with easily accessible media (eg, using infographics) to maximise international engagement. We will present findings via seminars to key health professionals, including Public Health England and Public Health Wales, health service commissioners, local authorities and government planning officials to make recommendations for future policy decisions in this area and to those who have an interest in improving GBS, CMD and promoting SWB. We also plan to publish papers in internationally peer-reviewed journals to disseminate the research to the wider academic community and add to the evidence base. We will share our results at national and internationally recognised conferences and promote our findings in academic circles.

## Supplementary Material

Reviewer comments

Author's manuscript
