# Supplementary material for: Longitudinal access and exposure to green-blue spaces and individual-level mental health and well-being: protocol for a longitudinal, population-wide record-linked natural experiment
Source: BMJ Open. 2019 Apr 20;9(4):e027289. doi: 10.1136/bmjopen-2018-027289 (PMC6528002; doi:10.1136/bmjopen-2018-027289)
Supplement: Author's manuscript [file bmjopen-2018-027289.draft_revisions.pdf]

BMJ Open is committed to open peer review. As part of this commitment we make the peer review history of every article we publish publicly available.

When an article is published we post the peer reviewers' comments and the authors' responses online. We also post the versions of the paper that were used during peer review. These are the versions that the peer review comments apply to.

The versions of the paper that follow are the versions that were submitted during the peer review process. They are not the versions of record or the final published versions. They should not be cited or distributed as the published version of this manuscript.

BMJ Open is an open access journal and the full, final, typeset and author-corrected version of record of the manuscript is available on our site with no access controls, subscription charges or pay-per-view fees (http://bmjopen.bmj.com).

If you have any questions on BMJ Open's open peer review process please email
info.bmjopen@bmj.com

BMJ Open

Longitudinal access and exposure to green-blue spaces and individual-level mental health and wellbeing: A study programme protocol for a longitudinal, population-wide record-linked natural experiment

Journal:	*BMJ Open*
Manuscript ID	bmjopen-2018-027289
Article Type:	Protocol
Date Submitted by the Author:	15-Oct-2018
Complete List of Authors:	Mizen, Amy; Swansea University, Heath Data Research-UK, Wales and Northern Ireland<br>Song, Jiao; Swansea University, Heath Data Research-UK, Wales and Northern Ireland<br>Fry, Richard; Swansea University, Heath Data Research-UK, Wales and Northern Ireland; Swansea University, National Centre for Population Health & Wellbeing Research<br>Akbari, Ashley; Swansea University, Heath Data Research-UK, Wales and Northern Ireland<br>Berridge, Damon; Swansea University, Heath Data Research-UK, Wales and Northern Ireland<br>Johnson, Rhodri; Swansea University, Heath Data Research-UK, Wales and Northern Ireland<br>Rebecca Lovell, Rebecca; University of Exeter Medical School, European Centre for Environment and Human Health<br>Lyons, Ronan; Swansea University, Heath Data Research-UK, Wales and Northern Ireland<br>Nieuwenhuijsen, Mark; Instituto de Salud Global de Barcelona (ISGlobal)<br>Stratton, Gareth; Swansea University, Heath Data Research-UK, Wales and Northern Ireland<br>Wheeler, Benedict; University of Exeter Medical School, European Centre for Environment and Human Health<br>White, James; Cardiff University, DECIPHer, Centre for Trials Research<br>White, Mathew; University of Exeter Medical School, European Centre for Environment and Human Health<br>Rodgers, Sarah; University of Liverpool, Department of Public Health and Policy; Swansea University, Heath Data Research-UK, Wales and Northern Ireland
Keywords:	Wellbeing, Environmental exposure, Geographic Information Systems, Routine linked data, Longitudinal, MENTAL HEALTH

SCHOLARONE™
Manuscripts

Longitudinal access and exposure to green-blue spaces and individual-level mental health and wellbeing: A study programme protocol for a longitudinal, population-wide record-linked natural experiment

Amy Mizen[1], Jiao Song[1], Richard Fry[1,2], Ashley Akbari[1], Damon Berridge[1], Rhodri Johnson[1], Rebecca Lovell[3], Ronan A Lyons[1], Mark Nieuwenhuijsen[4], Gareth Stratton[1], Benedict W. Wheeler[3], James White[5], Mathew White[3], Sarah Rodgers[1,6*]

1. Heath Data Research-UK, Wales and Northern Ireland, Swansea University, Singleton Park, Swansea, UK; 2. National Centre for Population Health & Wellbeing Research, Swansea University, Singleton Park, Swansea, UK; 3. European Centre for Environment and Human Health, University of Exeter Medical School, Knowledge Spa, Royal Cornwall Hospital, Truro, Cornwall, UK 4. Instituto de Salud Global de Barcelona.c/ Rosselló, 132, 5º 2ª (08036), Barcelona, Spain 5. DECIPHer, Centre for Trials Research, Cardiff University, 4th floor Neaudd Merinionydd, Heath, Cardiff, UK 6. Department of Public Health and Policy, University of Liverpool, Liverpool, UK

*Correspondence to: Professor Sarah E Rodgers, Department of Public Health and Policy, University of Liverpool, Liverpool, UK; Sarah.Rodgers@Liverpool.ac.uk

AM and JS contributed equally to this paper.

Keywords

Mental health; Wellbeing; Environmental exposure; Geographic Information Systems; Routine linked data; Longitudinal

Abstract

Introduction: Studies suggest that access and exposure to green-blue spaces (GBS) have beneficial impacts on mental health. However, the evidence base is limited with respect to longitudinal studies. The main aim of this longitudinal, population-wide, record-linked natural experiment, is to model the daily lived experience by linking GBS accessibility indices, residential GBS exposure and health data; to enable quantification of the impact of GBS on wellbeing and common mental health disorders, for a national population.

For peer review only - http://bmjopen.bmj.com/site/about/guidelines.xhtml

Methods and analysis: This research will estimate the impact of neighbourhood GBS access, GBS exposure and visits to GBS on the risk of common mental health conditions and the opportunity for promoting Subjective wellbeing (SWB); both key priorities for public health. We will use Geographical Information System (GIS) to create quarterly household GBS accessibility indices and GBS exposure using digital map and satellite data for 1.4 million homes in Wales, UK (2008-2018). We will link the GBS accessibility indices and GBS exposures to individual level mental health outcomes for 1.7 million people with General Practitioner (GP) data and data from the National Survey for Wales (n = ~12,000) on wellbeing in the Secure Anonymised Information Linkage (SAIL) databank. We will examine if these associations are modified by multiple socio-physical variables, migration and socioeconomic disadvantage. Subgroup analyses will examine associations by different types of GBS. This longitudinal study will be augmented by cross-sectional research using survey data on self-reported visits to GBS and SWB.

Ethics and dissemination:

All data will be anonymised and linked within the privacy protecting SAIL Databank. We will be using anonymised data and therefore we are exempt from National Research Ethics Committee (NREC). An Information Governance Review Panel (IGRP) application [Project ID: 0562] to link these data has been approved.

The research programme will be undertaken in close collaboration with public/patient involvement groups. A multi-strategy programme of dissemination is planned with the academic community, policy makers, practitioners and the public.

For peer review only

Strengths and limitations of this study

- *This retrospective controlled evaluation of a natural experiment includes every adult in Wales, UK between 2008-2018, minimising selection bias*
- *Generating a national longitudinal dataset of changes in GBS exposure and access to GBS for households will reduce ecological fallacy*
- *Spatial and temporal accessibility data linked for individuals and their routinely captured health service use, together with potential confounders, will allow us to investigate the impact of GBS on wellbeing*

For peer review only - http://bmjopen.bmj.com/site/about/guidelines.xhtml

- *Detailed self-report data on wellbeing and GBS visit behaviour from cross-sectional national survey data will be linked to health service utilisation data*
- *Despite including a large number of potentially confounding variables, this non-randomised study using routinely recorded data may omit some unknown confounders, thereby introducing a moderate level of bias due to confounding*

For peer review only

For peer review only - http://bmjopen.bmj.com/site/about/guidelines.xhtml

Introduction

Globally, 686 million people suffer with common mental health disorders (CMD) such as depression or anxiety [1]. In the UK, CMD are experienced by around 1 in 4 of the population, and mental ill-health costs the economy over £100bn pa in health, social care and quality of life-loss costs [2,3]. Subjective wellbeing (SWB) is also related to mental and physical health outcomes, including life expectancy [4,5], and is a key marker of quality of life [6]. With increasing impacts on wider societal costs, CMD and promoting SWB are growing in importance. Access to natural environments – considered here as 'green-blue spaces' (GBS) – such as parks and beaches – may provide opportunities to support and promote good public mental health and wellbeing [7,8]. The evidence base on the impacts of GBS, on mental health and wellbeing is growing rapidly. Current research suggests that the benefits may differ by population group, context and health outcome [9,10].

Recent systematic reviews, of predominantly cross-sectional studies, indicate positive relationships between mental health and SWB outcomes with living near GBS [11,12]. Access to GBS is positively associated with GBS use, and using GBS may improve health outcomes through a number of mechanisms. For example, increased physical activity [13,14], psychological restoration [15,16], noise mitigation [17], heat and humidity regulation [18,19], increased social interaction and cohesion [20–22]. Contrary to these findings, a study in New Zealand found no evidence that GBS influenced cardiovascular disease mortality [23] and suggested that GBS and health relationships may vary according to country or environmental contexts.

Many studies have found that access to GBS can vary across socio-economic status (SES) areas; with more deprived areas tending to have poorer access to GBS [24,25]. Differences in the distribution of GBS across SES may influence and contribute to SES-related health inequalities [26]. Furthermore, individuals from higher SES groups are more likely to select living in greener neighbourhoods [27]. Previous studies lack censoring for births, deaths and migration, which may have led to health selection effects which can create/over/underestimate the strength of association. There are also a lack of large-scale population level studies which have systematically and explicitly examined associations within and between sub-groups

For peer review only - http://bmjopen.bmj.com/site/about/guidelines.xhtml

other than SES [10] (e.g. by gender [28–30], age, education [22,31]). Furthermore, ethnic minority groups are underrepresented in studies due to selective non-response [32]. Studies that have focussed on differences between ethnic minorities found positive effects of GBS on wellbeing differed by ethnicity [33] and ethnic minorities had poorer access to GBS [34].

Study methods used to define access to GBS differ; some studies have calculated small area-level metrics and others have used distances. There is no accepted method to define GBS or for measuring different types of GBS (e.g. according to general landcover categories, ecosystems or landscape classifications) [35–41]. However, it is difficult to expect an authoritative voice on defining GBS access because how people engage with GBS varies by population subgroup and environmental context. Therefore, how GBS is defined will vary by study design and the focus of the research. We propose to take an approach that will consider land use but also access points, rights of way and amenities such as benches. There are also differences in study aims because of differing perspectives; such as health research, environmental research and policy. Few studies have also considered the issue of access to blue spaces [42,43] which has also contributed to diversity in GBS accessibility measures. Although there is now a significant body of research, there are few longitudinal studies on mental health outcomes in response to changes in GBS access or with assessments of actual visits to GBS [44–46].

We define *GBS accessibility* indices as metrics that represent how people may access GBS (Figure 1). *GBS exposure* is the ambient environment that people experience because of where they live (Figure 1). By including both of these measures in our study, we will be able to build a more holistic view of how GBS impacts CMD and wellbeing.

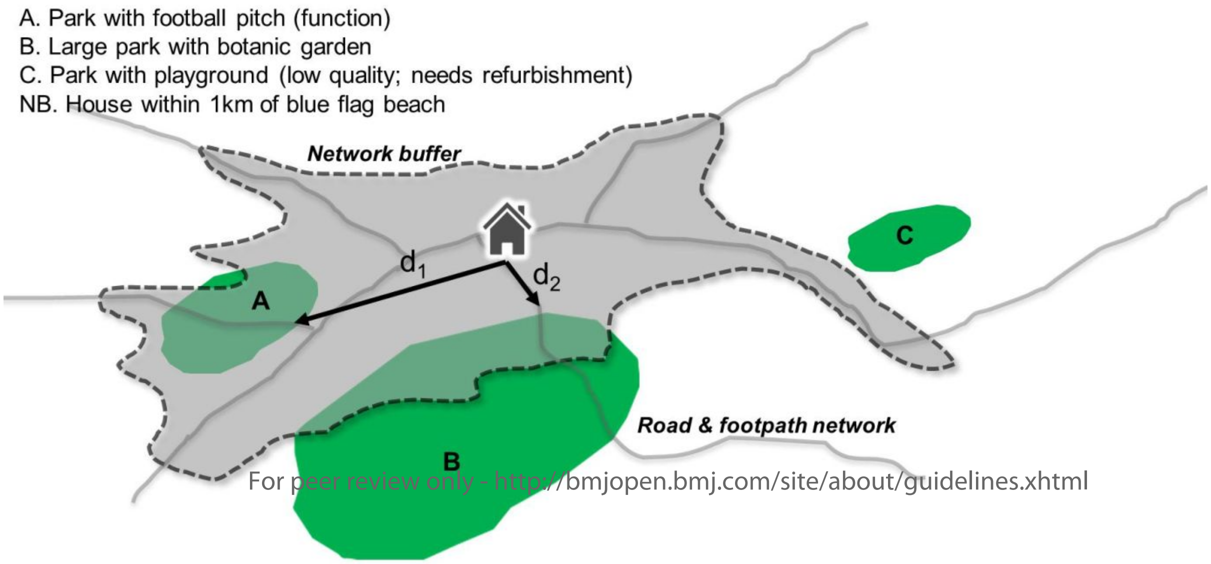

Figure 1.

For peer review only - http://bmjopen.bmj.com/site/about/guidelines.xhtml

Residential GBS access and exposure for each home 1) Household exposure defined as greenness immediately surrounding home/what can be seen out of the window. 2) Network buffer defines household access boundary (e.g. 10-minute walk). 3) GBS A and B included in access estimates including access points, rights of way and facilities such as benches (GBS C excluded). 3) Access measures include GBS quality, size, function and weighted network distances (d_i) to access points. 4) Longitudinal exposure estimates created through quarterly repeat analysis.

Aim

Our primary aim is to quantify the impact of changes in access to GBS and ambient GBS exposure on CMD and SWB, for a national population. This will be fulfilled through a number of objectives within two work packages. Work package 1 (WP1) will use routinely collected health and demographic data (data collected for purposes other than research [47]) stored in an anonymised databank to examine the risk of CMD using longitudinal changes in access to neighbourhood GBS. Work package 2 (WP2) will link routine data in the anonymised databank with in-depth survey responses from the data linked National Survey for Wales (NSW) to evaluate a representative sample of Welsh residents' self-reported wellbeing and GBS use.

Objectives

WP1

1. Create a longitudinal dataset of GBS in Wales using UK Ordnance Survey, Local Authority and remotely sensed satellite data;
2. Create longitudinal, residential GBS accessibility indices for all homes in Wales using the longitudinal GBS dataset in a GIS.
3. Create an 11-year dynamic cohort of individual-level longitudinal residential GBS accessibility indices to answer the research question(s): "*Do people with different GBS access through time have different associated risks of having a CMD?"*" and "*Is the association between changes in access to GBS and CMD modified by multiple socio-physical variables, migration and socioeconomic disadvantage?*"

WP2

1. Create data linkages between survey and routinely collected data within the Secure Anonymised Information Linkage databank (SAIL) for household-level GBS accessibility indices, and individual-level health and demographic data
2. Compliment residential GBS accessibility indices by including GBS usage from the data-linked National Survey for Wales to model interactions to answer research

For peer review only - http://bmjopen.bmj.com/site/about/guidelines.xhtml

question: "*Is the association between GBS residential access, modified by GBS use and multiple socio-physical modifiers, on wellbeing, and common mental health disorders (CMD)*?"

Methods and analysis

Design

The GBS project is a retrospective and controlled, population-wide study. We will evaluate the association between changes in access to GBS, on the risk of CMD (WP1) and SWB (WP2). This could be either at environment level (e.g. a change in the GBS itself) or person level (e.g. moving home, better access to transport). WP1 will use longitudinal data to examine variability in time and space of access to GBS and examine whether this could be due to planning and environmental policies. WP2 will use cross-sectional data to investigate whether visits to GBS improve SWB.

Participants

Our study population are made up of several different groups who are aged 16 years and older living in Wales, UK. WP1 includes the total adult population registered with a general practitioner (GP), providing GP records to SAIL. This is 1.7 million adults in Wales (see Figure 2). Dates for each update of the address record in a population register provide durations of residency for each successive address. WP2 includes a representative sample of the adult population in Wales based on the NSW for two years (cross-sectional samples in 2016/17 and 2017/18). The NSW has an annual sample of approximately 12,000 responders and GBS visit questions are asked of 50% of that sample. This provides a total cross-sectional sample of 12,000 (over the two years) for this part of the study.

For peer review only - http://bmjopen.bmj.com/site/about/guidelines.xhtml

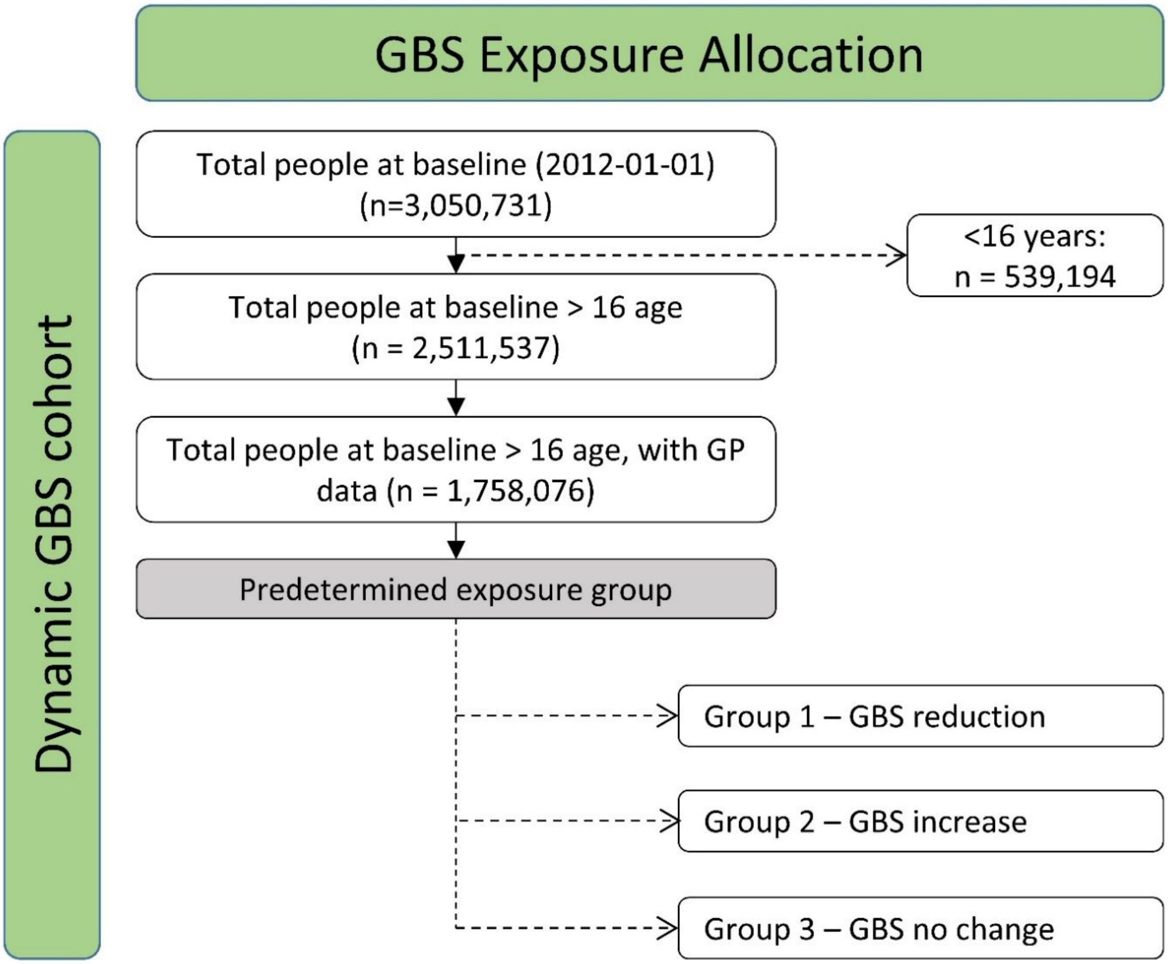

Figure 2. Flow diagram of proposed number (1.7 million adults) we currently have before exposure group allocation.

Research Environment

Environment data will be collected, stored and processed to generate household level GBS accessibility indices. This work will be undertaken in a secure GIS platform which is hosted by UKSeRP [48]. High resolution map data from the Ordnance Survey (OS) is already stored in the GIS, including point data for all residences (Address Base Premium (OS ABP)) and road network data [49,50].

In parallel with, but separate from the GIS, is the Secure Anonymised Information Linkage (SAIL) Databank [51–53]. Data linkage and statistical analysis for this study will be conducted within the SAIL Databank. The SAIL Databank contains longitudinal health, social and

For peer review only - http://bmjopen.bmj.com/site/about/guidelines.xhtml

education data on more than 5 million people which includes the active population of Wales, UK (3.1 million people) at any one time. The databank includes over 15 billion anonymised records and was designed to overcome data sharing issues.

A strength of the SAIL platform is the method for anonymising all individuals and households in Wales. Data are anonymised through the split-file process [51,52]. Whereby the dataset are separated in to identifiable (e.g. address) and non-identifiable (GBS access and exposure) components. The identifiable component is transported to our Trusted Third Party (TTP), NHS Wales Informatics Service (NWIS) [54]. The non-identifiable component is sent securely to the SAIL databank. The TTP anonymise and encrypt the identifiable data and each individual record is assigned a unique linking field. An Anonymous Linking Field (ALF) is assigned to individual level data and a Residential Anonymous linking Field (RALF) is assigned to a place of residence [54]. The anonymised elements of the dataset are then sent to the SAIL databank to be loaded. These elements are then recombined with the non-identifiable (GBS access and exposure) component of the dataset, which makes them ready for linkage with other datasets in the SAIL databank (see Figure 3).

Data Sources

Environment Datasets

We will create a longitudinal, national dataset of GBS for Wales (2008-2018) using temporally and spatially referenced satellite imagery [55]. We will also develop a typology of GBS and collate data from multiple sources to augment the all-Wales OS data we hold in our GIS database (OS Mastermap) [49,50,56]. Using satellite imagery, we will extract temporal trends of ‘greenness’ (using measures such as the Normalised Difference Vegetation Index (NDVI), which has been used in previous studies e.g. [57]) for every household in Wales and we will also explore the integration of new GBS and land use data as they become available and are published (e.g. OS Greenspace data [58]) and local authority audits of GBS.

For peer review only - http://bmjopen.bmj.com/site/about/guidelines.xhtml

GBS Accessibility and Exposure Dataset

There is no clear consensus in the existing literature on the most appropriate measures of GBS access and/or exposure [42]. Using the aforementioned Environment datasets, this project will collate a set of measures using criteria including longitudinal consistency, spatial resolution and evidence for associations with mental health and SWB outcomes. We will create residential level exposure measures by modelling the ambient home environment i.e. the GBS immediately surrounding their home and what they see out of their window. This metric will include measures such as greenness (NDVI), urban trees and garden size. The access measures will represent people's potential to visit GBS in their locality. We will adapt a previous methodology that modelled 'change in alcohol outlet density and alcohol-related harm to population health' (CHALICE) [59]. The distance decay for this accessibility model will be updated to emulate how people engage with GBS as opposed to alcohol outlets. People will behave differently in accessing GBS, compared with how they may access alcohol outlets. For example, people may be prepared to walk for a longer time and/or distance to access a GBS compared with alcohol outlets [60]. Our modelling will consider differential associations for key subgroups (e.g. deprived populations) thereby minimising risks that recommendations could increase inequalities. We will create a number of accessibility and exposure measures, allowing planners to consider the configuration (size, function, quality) of the most beneficial mix of spaces. We will also measure, for each adult, change in GBS access and exposure for the study period. Again, we will build on the CHALICE study [61] that measured change in alcohol outlet density by calculating change in density between the current and previous quarter and also the change between each quarter and baseline.

The longitudinal, household level, GBS accessibility and exposure dataset will be imported in to the SAIL Databank and linked with health datasets that are held within the SAIL Databank. Household identifiers (Unique Property Reference Numbers (UPRNs)) will be will be used to distinguish each household in the GIS and attached to each GBS accessibility and exposure index. Household identifiers will be replaced with an anonymised linking field within SAIL [62] (see Figure 3).

For peer review only - http://bmjopen.bmj.com/site/about/guidelines.xhtml

Health Datasets

Welsh Longitudinal General Practice (WLGP)

The Welsh Longitudinal General Practice (WLGP) dataset contains individual-level health data including Read codes for all diagnoses, symptoms and treatments recorded by a general practitioner (GP) for each person [63] from 2008-2018.

Welsh Demographic Service (WDS)

The Welsh Demographic Service (WDS) dataset contains addresses for all individuals who register with a GP. Dates for each address record update are recorded, thereby providing durations of residency for multiple homes and the ability to link to local environment indices at each time point. This dataset holds demographic data including age and gender. These data will be used to create population sub-groups based on age, gender and location for each period. The WDS contains historical patient provided address information linked anonymously at the individual level (the ALF) which is the primary key variable for record-linkage [51,52].

National Survey for Wales (NSW)

The National Survey for Wales (NSW) is a cross-sectional, representative sample of adult participants across Wales. Participants answer questions on public services such as education, transport, leisure activities, and self-reported health and well-being. They also report information on visits to GBS, such as visit frequency and activities undertaken during visits.

Figure 3 shows how GBS accessibility indices will be generated and linked with routinely collected health and survey data held within the SAIL databank. Table 1 shows the data sources and variables that we plan to derive from the routinely collected health data, survey dataset and population register that are available in the SAIL Databank.

For peer review only - http://bmjopen.bmj.com/site/about/guidelines.xhtml

For peer review only

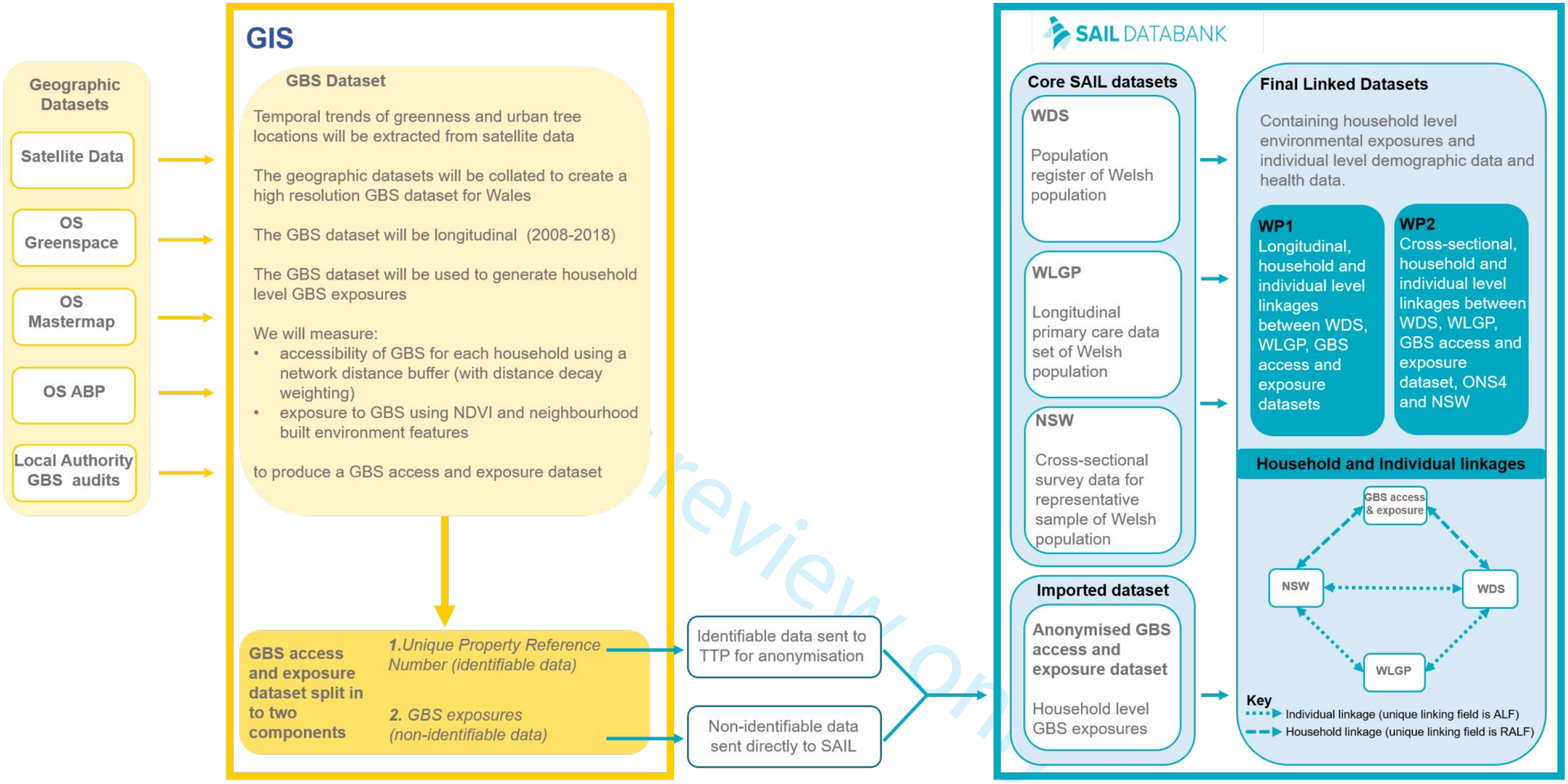

Figure 3: Diagram of proposed GIS data preparation and data linkage in SAIL Databank. GBS accessibility and exposure indices created within a secure GIS platform will be linked at household level with demographics from Welsh Demographic Service (WDS) and National Survey for Wales (NSW). Common mental health conditions from GP records will be linked at individual level with the NSW and WDS. Linkage will be undertaken by our Trusted Third Party (NHS Wales Informatics Service – NWIS).

For peer review only - http://bmjopen.bmj.com/site/about/guidelines.xhtml

Table 1. Data sources for outcome and accessibility/exposure variables to be used in statistical analysis for years 2008-2018

Dataset name	Data Source	Derived Variables	Coverage	Work package
GBS Accessibility and Exposure dataset	Local Authorities, Ordnance Survey, Satellite data sources	Typology to be developed. The dataset will include GBS such as parks, woodland, sports facilities, trees in residential areas	Wales	WP1 & WP2
Welsh Longitudinal General Practice (WLGP) dataset (core SAIL dataset)	Primary care records recorded by GP	CMDs such as anxiety and depression (see outcome variable section for details)	70% of practices in Wales provide data to SAIL	WP1 & WP2
Welsh Demographic Service dataset (core SAIL dataset)	NHS Wales Informatics Service	Age, gender, week of birth, multiple move in and out of home dates	Total population of Wales who are registered with a General Practitioner, a free to use service in the UK.	WP1 & WP2
National Survey for Wales (NSW) (restricted SAIL dataset)	Welsh Government	Subjective wellbeing, self-reported visits to GBS	Cross-sectional, representative sample approximately 12,000 participants per year	WP2

For peer review only - http://bmjopen.bmj.com/site/about/guidelines.xhtml

Statistical analysis

Outcome variables

(1) WP1 & 2: Primary Outcome – Common Mental Health Disorder (CMD)

Change in counts of CMD treatments for adults identified as CMD cases within the corresponding time periods for the 70% of adults in Wales for whom we have GP data records in SAIL (1.7M adults). Prevalence algorithms [64,65], detect cases of CMD (anxiety and depression) from routinely collected GP data. We will use the algorithm that incorporates historical diagnosis currently treaded with current diagnosis whether treated or untreated to identify CMD cases. For each adult, and for the time periods (quarters) identified as a CMD case, treatments will be counted per day and aggregated into quarterly counts. Figure 4 shows a conceptual model of exposure variable (GBS access and exposure) and primary outcome variable (CMD treatments).

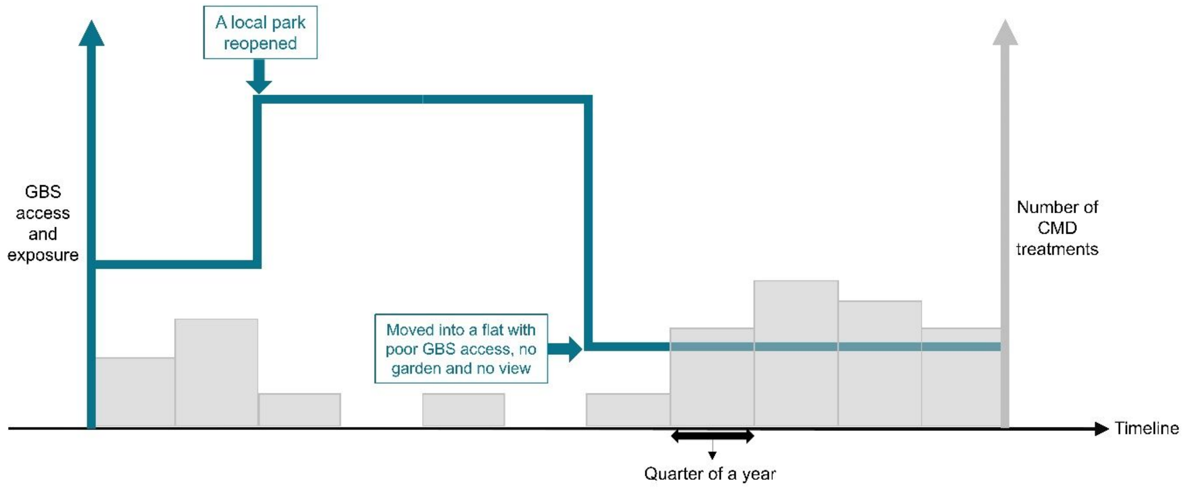

Figure 4: Conceptual model of primary exposure variable and primary outcome variable.

(2) WP2: Secondary Outcome – Subjective Wellbeing (SWB)

SWB is measured by the Warwick-Edinburgh Mental Wellbeing Scale (WEMWBS) in the NSW for two survey years (2016/17 and 2017/18) for a representative population [66]. The WEMWBS is comprehensive (incorporating elements of both subjective and psychological wellbeing), short enough to be used in population-

For peer review only - http://bmjopen.bmj.com/site/about/guidelines.xhtml

level surveys, responsive to change and has been validated among community samples of adults in the UK [67]. WEMWBS scores are on continuous scale from 14 to 70.

(3) WP1: Secondary outcome – GP events

We have the total number of events recorded for each person in a WLGP dataset. We will calculate the number of GP events, converting to a binary daily activity or no activity aggregated to quarterly counts. This eliminates double counting (e.g. counting the return of large quantities of test results only once). These are purposefully all events rather than those related only to CMD because this will capture people who frequently visit their GP but for whom no CMD diagnosis has been made. This will allow us to explore social isolation and as indicator of undiagnosed CMD.

(4) WP2: Secondary outcome – ONS Subjective Wellbeing

We will have individual responses in NSW to the 4 standard SWB questions (life satisfaction, sense of worth of activities, happiness and anxiety) as used by Office for National Statistics and the Organisation for Economic Co-operation and Development [68].As well as being relatable to national representative survey data, these data have also been used in previous GBS visit research using the comparable Monitor of Engagement with the Natural Environment for England [45].

Analysis plan

We will provide descriptive statistics of GBS access and exposure and changes in GBS access and exposure by household, LSOA, deprivation quintiles using WIMD and population characteristics. National Survey for Wales data will have descriptive statistics of each of the survey questions we will use (number of visits, labour market status, etc.).

Across both work packages, we have outcomes of two distinct types: continuous (SWB) and counts (CMD treatments, and GP event days). The continuous outcome will be

For peer review only - http://bmjopen.bmj.com/site/about/guidelines.xhtml

intended to be analysed using a linear model. Poisson models will be used to analyse the longitudinal count data.

Our data are hierarchical in nature: WP1: three level data: LSOA/ Individual / Time (Quarter); WP2: two level data: LSOA/Individual. Accordingly, we will adopt a multilevel modelling approach. We will generalise the standard linear and Poisson models to handle two or three levels of variation, as appropriate. The resulting two- and three-level models will allow us to estimate the extent to which variation at each level may be explained by confounding variables (see Table 2). Having controlled for confounders, we will proceed to add access and exposure variables to our models, thereby allowing us to test for the significance of the access and exposure variables in the presence of the confounders. Inclusion of these variables in the above models will permit us to estimate their direct effects on our outcomes. Having included the main effects of both confounders and access and exposure variables, we will proceed to test for the significance of selected pairwise interactions of interest, as shown in Table 2.

For peer review only

For peer review only - http://bmjopen.bmj.com/site/about/guidelines.xhtml

Table 2. Analysis Summary (P: Primary; S: Secondary)

WP	Outcomes (Type)	Exposures	Confounders	Interactions of interest
**1**	P: CMD (count)	P: Access and exposure to GBS	LSOA level: Quintile of deprivation (WIMD). Category of urban/rural settlement type (ONS classification)	Change in access and exposure to GBS by deprivation
	S: GP event days (count)	S: Time of move(s);	Individual level (SAIL): Gender, Age, Comorbidities	Change in access and exposure to GBS by time of move(s)
**2**	P2: SWB (continuous)	P2: Access and exposure to GBS	LSOA level: as per WP1.	Exact number of visits by individual level deprivation
	P1: CMD (count)	S: Level of engagement in 150+ minutes of moderate or vigorous intensity activity per week	Individual level and NSW: Gender, Age, Comorbidities , Highest educational qualification, Marital status (incl. living with partner)<br><br>Exact number of visits made	Exact number of visits by residential GBS

For peer review only - http://bmjopen.bmj.com/site/about/guidelines.xhtml

		outdoors for recreation in last 4 weeks

For peer review only - http://bmjopen.bmj.com/site/about/guidelines.xhtml

For peer review only

Discussion

This study will be the first of its kind to link GIS-generated GBS accessibility and exposure measures with routinely collected, longitudinal health data and cross-sectional survey data for a whole population. We plan to create a longitudinal GBS dataset for Wales. Using multi-source data we will build a dataset that records local level changes in GBS for Wales, for 11 years. Longitudinal studies that have previously examined GBS exposure have used cross-sectional environment data to calculate GBS accessibility and exposure indices. This may be because it is a resource and time-intensive task to create a longitudinal GBS dataset, and longitudinal data is not always available. In addition, bringing together data from different sources, harmonising the data and deriving GBS indices from the data requires expertise and specialised skills.

Our study will work with stakeholders and policy makers to develop a GBS typology that can be used to provide evidence that can be translated to help policy and practice. Improved evidence on the impacts of GBS on mental health is required to inform decisions relating to planning, area regeneration and environmental management. Natural England, England's statutory body responsible for the natural environment, concluded that a knowledge of causal pathways and contributory mechanisms that link mental health and environmental exposure is required [69]. This study will produce the evidence needed to address gaps stated by the Welsh Government Environment Bill White Paper [70] and inform implementation of the Well-being of Future Generations (Wales) Act 2015 [71]. We have key policy makers signed up as stakeholders for this project and they will be involved in discussions for how we define GBS, how we measure access and exposure to GBS and in the reporting of our results so that this study can be useful in generating evidence-based policy.

Another aim of the study is to create novel GBS accessibility indices and that will model GBS exposure in way that reflects how people may access GBS. The way that studies measure access to GBS is methodologically diverse and there is no general consensus on what is the best measure to use. Our measures will be informed by theories and findings in the literature and to compliment this, we will invite members of the public to

For peer review only - http://bmjopen.bmj.com/site/about/guidelines.xhtml

workshops and ask them to help direct our research through a series of focussed questions. The workshop group will comprise members who are experienced at considering the value of environment from the Health and Environment Public Engagement (HEPE) Group (hosted by University of Exeter), who will join Wales-based members of the public from urban park groups, and those experienced in considering data linkage proposals [72].

The overarching aim of our longitudinal study is to identify causal mechanisms to determine whether a positive change in access and exposure to GBS lowers the risk of CMD.

Ethics and dissemination

Ethical arrangements

This study is based on routinely collected administrative, environment, and survey data. All data will be anonymised into a secure databank and therefore there will be no mechanism for informing potential study participants of possible benefits and known risks. We have obtained informed consent to use the linked and anonymised NSW data within the SAIL Databank. All routinely collected anonymised data held in SAIL, are exempt from consent due to the anonymised nature of the databank (under section 251, NREC).

Research Governance

We have applied and been granted approval by the independent Information Governance Review Panel (IGRP) for permission to conduct this study (study number 0562). The IGRP contains independent members from NREC and BMA, as well as lay members, and have previously given permission for similar projects (e.g. NIHR PHR CHALICE and NIHR PHR Carmarthenshire Housing). The review process has checked that the study we are is useful, not service evaluation, and will not break anonymisation standards.

For peer review only - http://bmjopen.bmj.com/site/about/guidelines.xhtml

Dissemination Policy

We will regularly report our progress to the Study Steering Committee (SSC). The SSC will comprise academic experts and stakeholders (NRW, Keep Wales Tidy, Sport Wales and Welsh Government). At the end of the study we will hold a workshop to report our findings to stakeholders and the public. We will disseminate our findings to patient, policy and academic networks (e.g. Health Data Research-UK, Administrative Data Research and National Institute for Health Research) and we will present results to the public with easily accessible media (e.g. using infographics) to maximise international engagement. We will present findings via seminars to key health professionals, including Public Health England and Public Health Wales, health service commissioners, Local Authorities and government planning officials to make recommendations for future policy decisions in this area and to those who have an interest in improving GBS, CMD and promoting SWB. We also plan to publish papers in internationally peer reviewed journals to disseminate the research to the wider academic community and add to the evidence base. We will share our results at national and internationally recognised conferences and promote our findings in academic circles.

Abbreviations

ABP	Address Base Premium
BMA	British Medical Association
CHALICE	Change in alcohol outlet density and alcohol-related harm to population health
CMD	Common Mental Health Disorders
GBS	Green-Blue Spaces
GIS	Geographic Information Systems
GP	General Practitioner
HEPE	Health and Environment Public Engagement
IGRP	Information Governance Review Panel
LSOA	Local Super Output Area
NDVI	Normalised Difference Vegetation Index
NREC	National Research Ethics Committee
NRW	Natural Resources Wales
NSW	National Survey for Wales
ONS	Office for National Statistics

For peer review only - http://bmjopen.bmj.com/site/about/guidelines.xhtml

OS	Ordnance Survey
SAIL	Secure Anonymised Information Linkage
SSC	Study Steering Committee
SWB	Subjective Wellbeing
TTP	Trusted Third Party
UKSeRP	United Kingdom Secure e-Research Platform
UPRN	Unique Property Reference Number
WDS	Welsh Demographic Service
WEMWBS	Warwick-Edinburgh Mental Wellbeing Scale
WIMD	Welsh Index of Multiple deprivation
WLGP	Welsh Longitudinal General Practice
WP1	Work Package 1
WP2	Work Package 2

For peer review only

For peer review only - http://bmjopen.bmj.com/site/about/guidelines.xhtml

References

1 World Health Organization. Depression and other common mental disorders: global health estimates. *World Heal Organ* 2017;:1–24. doi:CC BY-NC-SA 3.0 IGO

2 Davies S. Annual Report of the Chief Medical Officer 2013. Public Mental Health Priorities: Investing in the Evidence. London: : Department of Health 2013.

3 Centre For Mental Health | Economic and social costs of mental health problems. https://www.centreformentalhealth.org.uk/economic-and-social-costs (accessed 13 Jun 2018).

4 Diener E, Chan MY. Happy People Live Longer: Subjective Well-Being Contributes to Health and Longevity. *Appl Psychol Heal Well-Being* 2011;**3**:1–43. doi:10.1111/j.1758-0854.2010.01045.x

5 Steptoe A, Deaton A, Stone AA. Subjective wellbeing, health, and ageing. *Lancet* 2015;**385**:640–8. doi:10.1016/S0140-6736(13)61489-0

6 WHO. The World Health Organization Quality of Life assessment (WHOQOL): position paper from the World Health Organization. *Soc Sci Med* 1995;**41**:1403–9. doi:10.1016/0277-9536(95)00112-K

7 Hartig T, Mitchell R, de Vries S, *et al.* Nature and Health. *Annu Rev Public Health* 2014;**35**:207–28. doi:10.1146/annurev-publhealth-032013-182443

8 Marmot M. Fair society, healthy lives. 2010.

9 Markevych I, Schoierer J, Hartig T, *et al.* Exploring pathways linking greenspace to health: Theoretical and methodological guidance. *Environ Res* 2017;**158**:301–17. doi:10.1016/j.envres.2017.06.028

10 Dadvand P, de Nazelle A, Figueras F, *et al.* Green space, health inequality and pregnancy. *Environ Int* 2012;**40**:110–5. doi:10.1016/j.envint.2011.07.004

11 Bowler DE, Buyung-Ali LM, Knight TM, *et al.* A systematic review of evidence for the added benefits to health of exposure to natural environments. *BMC Public Health* 2010;**10**:456. doi:10.1186/1471-2458-10-456

12 James P, Banay RF, Hart JE, *et al.* A Review of the Health Benefits of Greenness. *Curr Epidemiol Reports* 2015;**2**:131–42. doi:10.1007/s40471-015-0043-7

For peer review only - http://bmjopen.bmj.com/site/about/guidelines.xhtml

13 Thompson Coon J, Boddy K, Stein K, *et al.* Does participating in physical activity in outdoor natural environments have a greater effect on physical and mental wellbeing than physical activity indoors? A systematic review. *Environ Sci Technol* 2011;**45**:1761–72. doi:10.1021/es102947t

14 Coombes E, Jones AP, Hillsdon M. The relationship of physical activity and overweight to objectively measured green space accessibility and use. *Soc Sci Med* 2010;**70**:816–22. doi:10.1016/j.socscimed.2009.11.020

15 White MP, Alcock I, Wheeler BW, *et al.* Would You Be Happier Living in a Greener Urban Area? A Fixed-Effects Analysis of Panel Data. *Psychol Sci* 2013;:0956797612464659. doi:10.1177/0956797612464659

16 White MP, Alcock I, Wheeler BW, *et al.* Coastal proximity, health and well-being: Results from a longitudinal panel survey. *Heal Place* 2013;**23**:97–103. doi:10.1016/j.healthplace.2013.05.006

17 Hystad P, Davies HW, Frank L, *et al.* Residential Greenness and Birth Outcomes: Evaluating the Influence of Spatially Correlated Built-Environment Factors. *Environ Health Perspect* 2014;**122**:1095–102. doi:10.1289/ehp.1308049

18 Dadvand P, Sunyer J, Basagaña X, *et al.* Surrounding greenness and pregnancy outcomes in four Spanish birth cohorts. *Environ Health Perspect* 2012;**120**:1481–7. doi:10.1289/ehp.1205244

19 Kingsley SL, Eliot MN, Whitsel EA, *et al.* Maternal residential proximity to major roadways, birth weight, and placental DNA methylation. *Environ Int* 2016;**92–93**:43–9. doi:10.1016/j.envint.2016.03.020

20 De Vries S, van Dillen SME, Groenewegen PP, *et al.* Streetscape greenery and health: Stress, social cohesion and physical activity as mediators. *Soc Sci Med* 2013;**94**:26–33. doi:10.1016/j.socscimed.2013.06.030

21 van den Berg MM, van Poppel M, van Kamp I, *et al.* Do Physical Activity, Social Cohesion, and Loneliness Mediate the Association Between Time Spent Visiting Green Space and Mental Health? *Environ Behav* 2017;:001391651773856. doi:10.1177/0013916517738563

22 Maas J, van Dillen SME, Verheij RA, *et al.* Social contacts as a possible mechanism behind the relation between green space and health. *Heal Place*

For peer review only - http://bmjopen.bmj.com/site/about/guidelines.xhtml

2009;**15**:586–95. doi:10.1016/j.healthplace.2008.09.006

23 Richardson E, Pearce J, Mitchell R, *et al.* The association between green space and cause-specific mortality in urban New Zealand: an ecological analysis of green space utility. *BMC Public Health* 2010;**10**:240. doi:10.1186/1471-2458-10-240

24 Mitchell R, Astell-Burt T, Richardson EA. A comparison of green space indicators for epidemiological research. *J Epidemiol Community Health* 2011;**65**:853–8. doi:10.1136/jech.2010.119172

25 Mitchell R, Popham F. Effect of exposure to natural environment on health inequalities: an observational population study. *Lancet* 2008;**372**:1655–60. doi:10.1016/S0140-6736(08)61689-X

26 Sugiyama T, Villanueva K, Knuiman M, *et al.* Can neighborhood green space mitigate health inequalities? A study of socio-economic status and mental health. *Heal Place* 2016;**38**:16–21. doi:10.1016/j.healthplace.2016.01.002

27 Amoly E, Dadvand P, Forns J, *et al.* Green and blue spaces and behavioral development in barcelona schoolchildren: The BREATHE project. *Environ Health Perspect* 2015;**122**:1351–8. doi:10.1289/ehp.1408215

28 Astell-Burt T, Mitchell R, Hartig T. The association between green space and mental health varies across the lifecourse. a longitudinal study. *J Epidemiol Community Health* 2014;**68**:578–83. doi:10.1136/jech-2013-203767

29 Mitchell R, Popham F. Greenspace, urbanity and health: Relationships in England. *J Epidemiol Community Health* 2007;**61**:681–3. doi:10.1136/jech.2006.053553

30 Reklaitiene R, Grazuleviciene R, Dedele A, *et al.* The relationship of green space, depressive symptoms and perceived general health in urban population. *Scand J Public Health* 2014;**42**:669–76. doi:10.1177/1403494814544494

31 de Vries S, Verheij RA, Groenewegen PP, *et al.* Natural environments - Healthy environments? An exploratory analysis of the relationship between greenspace and health. *Environ Plan A* 2003;**35**:1717–31. doi:10.1068/a35111

32 van den Berg M, Wendel-Vos W, van Poppel M, *et al.* Health benefits of green spaces in the living environment: A systematic review of epidemiological studies.

For peer review only

For peer review only - http://bmjopen.bmj.com/site/about/guidelines.xhtml

Urban For Urban Green 2015;**14**:806–16. doi:10.1016/j.ufug.2015.07.008

33 McEachan RRC, Yang TC, Roberts H, *et al.* Availability, use of, and satisfaction with green space, and children's mental wellbeing at age 4 years in a multicultural, deprived, urban area: results from the Born in Bradford cohort study. *Lancet Planet Heal* 2018;**2**:e244–54. doi:10.1016/S2542-5196(18)30119-0

34 Dai D. Racial/ethnic and socioeconomic disparities in urban green space accessibility: Where to intervene? *Landsc Urban Plan* 2011;**102**:234–44. doi:10.1016/j.landurbplan.2011.05.002

35 Coolen H, Meesters J. Private and public green spaces: Meaningful but different settings. *J Hous Built Environ* 2012;**27**:49–67. doi:10.1007/s10901-011-9246-5

36 Taylor L, Hochuli DF. Defining greenspace: Multiple uses across multiple disciplines. *Landsc Urban Plan* 2017;**158**:25–38. doi:10.1016/j.landurbplan.2016.09.024

37 Wheeler BW, Lovell R, Higgins SL, *et al.* Beyond greenspace: an ecological study of population general health and indicators of natural environment type and quality. *Int J Health Geogr* 2015;**14**:17. doi:10.1186/s12942-015-0009-5

38 Alcock I, White MP, Lovell R, *et al.* What accounts for 'England's green and pleasant land'? A panel data analysis of mental health and land cover types in rural England. *Landsc Urban Plan* 2015;**142**:38–46. doi:10.1016/j.landurbplan.2015.05.008

39 MacKerron G, Mourato S. Happiness is greater in natural environments. *Glob Environ Chang* 2013;**23**:992–1000. doi:10.1016/j.gloenvcha.2013.03.010

40 van Dillen SME, de Vries S, Groenewegen PP, *et al.* Greenspace in urban neighbourhoods and residents' health: Adding quality to quantity. *J Epidemiol Community Health* 2012;**66**:1–5. doi:10.1136/jech.2009.104695

41 Annerstedt M, Östergren P-O, Björk J, *et al.* Green qualities in the neighbourhood and mental health – results from a longitudinal cohort study in Southern Sweden. *BMC Public Health* 2012;**12**:337. doi:10.1186/1471-2458-12-337

42 Ekkel ED, de Vries S. Nearby green space and human health: Evaluating accessibility metrics. *Landsc Urban Plan* 2017;**157**:214–20. doi:10.1016/j.landurbplan.2016.06.008

For peer review only - http://bmjopen.bmj.com/site/about/guidelines.xhtml

43 Grellier J, White MP, Albin M, *et al.* BlueHealth: A study programme protocol for mapping and quantifying the potential benefits to public health and well-being from Europe's blue spaces. *BMJ Open* 2017;**7**:1–10. doi:10.1136/bmjopen-2017-016188

44 Gascon M, Triguero-Mas M, Martínez D, *et al.* Mental Health Benefits of Long-Term Exposure to Residential Green and Blue Spaces: A Systematic Review. *Int J Environ Res Public Health* 2015;**12**:4354–79. doi:10.3390/ijerph120404354

45 White MP, Pahl S, Wheeler BW, *et al.* Natural environments and subjective wellbeing: Different types of exposure are associated with different aspects of wellbeing. *Heal Place* 2017;**45**:77–84. doi:10.1016/j.healthplace.2017.03.008

46 White MP, Pahl S, Ashbullby K, *et al.* Feelings of restoration from recent nature visits. *J Environ Psychol* 2013;**35**:40–51. doi:10.1016/j.jenvp.2013.04.002

47 Nicholls SG, Langan SM, Benchimol EI. Routinely collected data: The importance of high-quality diagnostic coding to research. *Cmaj* 2017;**189**:E1054–5. doi:10.1503/cmaj.170807

48 Health Informatics Group SUMS. UK Secure eResearch Platform UKSeRP Data Sharing : Why Should We Share Data? Swansea: 2017. https://saildatabank.com/wp-content/uploads/UKSeRP_Brochure_v1.5.pdf

49 AddressBase Premium | Business and government | Ordnance Survey. http://www.ordnancesurvey.co.uk/business-and-government/products/addressbase-premium.html (accessed 10 Mar 2014).

50 Ordnance Survey. OS(R) MasterMap(R) Integrated Transportation NetworkTM Layer technical specification. (c) Crown copyright 2011. 2012.http://www.ordnancesurvey.co.uk/oswebsite/support/products/os-mastermap/itn-layer-technical-specification/index.html%5Cnhttp://www.ordnancesurvey.co.uk/oswebsite/docs/user-guides/points-of-interest-user-guide.pdf%5Cnhttp://www.ordnancesurvey.co.uk/oswebsi

51 Lyons R a, Jones KH, John G, *et al.* The SAIL databank: linking multiple health and social care datasets. *BMC Med Inform Decis Mak* 2009;**9**:3. doi:10.1186/1472-6947-9-3

For peer review only - http://bmjopen.bmj.com/site/about/guidelines.xhtml

52 Ford D V., Jones KH, Verplancke J-P, *et al.* The SAIL Databank: building a national architecture for e-health research and evaluation. *BMC Health Serv Res* 2009;**9**:157. doi:10.1186/1472-6963-9-157

53 Rodgers SE, Lyons RA, Dsilva R, *et al.* Residential Anonymous Linking Fields (RALFs): A novel information infrastructure to study the interaction between the environment and individuals' health. *J Public Health (Bangkok)* 2009;**31**:582–8. doi:10.1093/pubmed/fdp041

54 SAIL Databank - The Secure Anonymised Information Linkage Databank. https://saildatabank.com/saildata/data-privacy-security/#protecting-identities (accessed 3 Oct 2017).

55 Gan M, Deng J, Zheng X, *et al.* Monitoring urban greenness dynamics using multiple endmember spectral mixture analysis. *PLoS One* 2014;**9**. doi:10.1371/journal.pone.0112202

56 Ordnance Survey. OS MasterMap Topography Layer. 2017.https://www.ordnancesurvey.co.uk/business-and-government/products/topography-layer.html (accessed 15 Nov 2017).

57 Beyer KMM, Kaltenbach A, Szabo A, *et al.* Exposure to neighborhood green space and mental health: Evidence from the survey of the health of wisconsin. *Int J Environ Res Public Health* 2014;**11**:3453–72. doi:10.3390/ijerph110303453

58 OS Greenspace | OS GetOutside.

59 Fry R, Orford S, Rodgers S. A best practice framework to measure spatial variation in alcohol availability. *Environ Plan B Plan Des* 2018;**0**:1–19. doi:10.1177/2399808318773761

60 Fone D, Morgan J, Fry R, *et al.* Change in alcohol outlet density and alcohol-related harm to population health (CHALICE): a comprehensive record-linked database study in Wales. *Chang alcohol outlet density alcohol-related harm to Popul Heal a Compr Rec database study Wales* 2016;**4**. doi:10.3310/phr04030

61 Fone D, Dunstan F, Lee S, *et al.* Change in alcohol outlet density and alcohol-related harm to population health (CHALICE): A comprehensive record-linked database study in Wales. 2015.http://www.nets.nihr.ac.uk/projects/phr/09300702 (accessed 24 Jun 2015).

For peer review only - http://bmjopen.bmj.com/site/about/guidelines.xhtml

For peer review only

62 Rodgers SE, Lyons RA, Dsilva R, *et al.* Residential Anonymous Linking Fields (RALFs): A Novel Information Infrastructure to Study the Interaction between the Environment and Individuals' Health. *J Public Health (Bangkok)* 2009;**10**:1–7. doi:10.1093/pubmed/fdp041

63 Read Codes - NHS Digital. https://digital.nhs.uk/article/1104/Read-Codes (accessed 19 Dec 2017).

64 John A, McGregor J, Fone D, *et al.* Case-finding for common mental disorders of anxiety and depression in primary care: an external validation of routinely collected data. *BMC Med Inform Decis Mak* 2016;**16**:35. doi:10.1186/s12911-016-0274-7

65 Cornish RP, John A, Boyd A, *et al.* Defining adolescent common mental disorders using electronic primary care data: a comparison with outcomes measured using the CIS-R. *BMJ Open* 2016;**6**:e013167. doi:10.1136/bmjopen-2016-013167

66 Aumeyr M, Brown Z, Doherty R, *et al.* National Survey for Wales 2016-17 Technical Report (Updated) National Survey for Wales 2016-17 Technical Report. Published Online First: 2017.http://doc.ukdataservice.ac.uk/doc/8301/mrdoc/pdf/8301_171018-national-survey-wales-2016-17-technical-report-en.pdf (accessed 19 Jun 2018).

67 Tennant R, Hiller L, Fishwick R, *et al.* The Warwick-Edinburgh Mental Well-being Scale (WEMWBS): development and UK validation. *Health Qual Life Outcomes* 2007;**5**:63. doi:10.1186/1477-7525-5-63

68 Personal Well-being user guidance - Office for National Statistics. https://www.ons.gov.uk/peoplepopulationandcommunity/wellbeing/methodologies/personalwellbeingsurveyuserguide (accessed 30 Aug 2018).

69 Natural England. Links between natural environments and mental health: evidence briefing - EIN018. 2016. publications.naturalengland.org.uk/file/4681263907078l4

70 Welsh Government. White Paper Towards the Sustainable Management of Wales ' Natural Resources, Consultation on proposals for an Environmental Bill, WG19631. 2013.

71 Welsh Government | Well-being of Future Generations (Wales) Act 2015.

For peer review only - http://bmjopen.bmj.com/site/about/guidelines.xhtml

http://gov.wales/topics/people-and-communities/people/future-generations-act/?lang=en (accessed 7 Nov 2017).

72 SAIL Databank - Consumer Panel. https://saildatabank.com/about-us/public-engagement/ (accessed 12 Jul 2018).

Contributors

AM and JS contributed equally to writing this paper. This paper was based on a grant proposal developed by SR, RF, AA, DB, RJ, BW, RL, RL, MN, GS, BW, JW and MW. This paper was written based on a draft manuscript written by RF based on the grant proposal. Specifically, the Introduction, and Discussion was written by AM. JS wrote the Analysis plan and developed the conceptual model. The Methods section was written by AM and JS. All the co-authors made substantial contributions to the writing of the Methods section and editing of the whole paper. All authors then read the final version and approved it for submission and publication.

Funding

Funding acknowledgement

This project was funded by the NIHR PHR (project number 16/07/07)

Department of Health and Social Care disclaimer

The views expressed are those of the author(s) and not necessarily those of the NHS, the NIHR or the Department of Health and Social Care.

This work was supported by Health Data Research UK (NIWA1), which is funded by the UK Medical Research Council, Engineering and Physical Sciences Research Council, Economic and Social Research Council, National Institute for Health Research (England), Chief Scientist Office of the Scottish Government Health and Social Care Directorates, Health and Social Care Research and Development Division (Welsh Government), Public Health Agency (Northern Ireland), British Heart Foundation and Wellcome. This work was

For peer review only - http://bmjopen.bmj.com/site/about/guidelines.xhtml

supported by the National Centre for Population Health and Well-Being Research (NXPHWR).

Acknowledgement

This study makes use of anonymised data held in the Secure Anonymised Information Linkage (SAIL) Databank. We would like to acknowledge all the data providers who make anonymised data available for research.

Competing interests

None

For peer review only

For peer review only - http://bmjopen.bmj.com/site/about/guidelines.xhtml

STROBE Statement—checklist of items that should be included in reports of observational studies

	Item No.	Recommendation	Page No.
**Title and abstract**	1	(*a*) Indicate the study's design with a commonly used term in the title or the abstract	1
		(*b*) Provide in the abstract an informative and balanced summary of what was done and what was found	1-2
**Introduction**			
Background/rationale	2	Explain the scientific background and rationale for the investigation being reported	3-5
Objectives	3	State specific objectives, including any prespecified hypotheses	5-6
**Methods**			
Study design	4	Present key elements of study design early in the paper	6-12
Setting	5	Describe the setting, locations, and relevant dates, including periods of recruitment, exposure, follow-up, and data collection	6-12
Participants	6	(*a*) *Cohort study*—Give the eligibility criteria, and the sources and methods of selection of participants. Describe methods of follow-up<br>*Case-control study*—Give the eligibility criteria, and the sources and methods of case ascertainment and control selection. Give the rationale for the choice of cases and controls<br>*Cross-sectional study*—Give the eligibility criteria, and the sources and methods of selection of participants	6-12
		(*b*) *Cohort study*—For matched studies, give matching criteria and number of exposed and unexposed<br>*Case-control study*—For matched studies, give matching criteria and the number of controls per case	
Variables	7	Clearly define all outcomes, exposures, predictors, potential confounders, and effect modifiers. Give diagnostic criteria, if applicable	13-14
Data sources/ measurement	8*	For each variable of interest, give sources of data and details of methods of assessment (measurement). Describe comparability of assessment methods if there is more than one group	13-14
Bias	9	Describe any efforts to address potential sources of bias	2
Study size	10	Explain how the study size was arrived at	7

Continued on next page

For peer review only

Quantitative variables	11	Explain how quantitative variables were handled in the analyses. If applicable, describe which groupings were chosen and why	13-16
Statistical methods	12	(*a*) Describe all statistical methods, including those used to control for confounding	14-16
		(*b*) Describe any methods used to examine subgroups and interactions	14-16
		(*c*) Explain how missing data were addressed	N/A
		(*d*) *Cohort study*—If applicable, explain how loss to follow-up was addressed<br>*Case-control study*—If applicable, explain how matching of cases and controls was addressed<br>*Cross-sectional study*—If applicable, describe analytical methods taking account of sampling strategy	N/A
		(*e*) Describe any sensitivity analyses	N/A
**Results**			
Participants	13*	(a) Report numbers of individuals at each stage of study—eg numbers potentially eligible, examined for eligibility, confirmed eligible, included in the study, completing follow-up, and analysed	N/A
		(b) Give reasons for non-participation at each stage	N/A
		(c) Consider use of a flow diagram	N/A
Descriptive data	14*	(a) Give characteristics of study participants (eg demographic, clinical, social) and information on exposures and potential confounders	N/A
		(b) Indicate number of participants with missing data for each variable of interest	N/A
		(c) *Cohort study*—Summarise follow-up time (eg, average and total amount)	N/A
Outcome data	15*	*Cohort study*—Report numbers of outcome events or summary measures over time	N/A
		Case-control study—Report numbers in each exposure category, or summary measures of exposure	N/A
		Cross-sectional study—Report numbers of outcome events or summary measures	
Main results	16	(*a*) Give unadjusted estimates and, if applicable, confounder-adjusted estimates and their precision (eg, 95% confidence interval). Make clear which confounders were adjusted for and why they were included	N/A
		(*b*) Report category boundaries when continuous variables were categorized	N/A
		(*c*) If relevant, consider translating estimates of relative risk into absolute risk for a meaningful time period	N/A

Continued on next page

For peer review only

Other analyses	17	Report other analyses done—eg analyses of subgroups and interactions, and sensitivity analyses	N/A
**Discussion**			
Key results	18	Summarise key results with reference to study objectives	N/A
Limitations	19	Discuss limitations of the study, taking into account sources of potential bias or imprecision. Discuss both direction and magnitude of any potential bias	17-18
Interpretation	20	Give a cautious overall interpretation of results considering objectives, limitations, multiplicity of analyses, results from similar studies, and other relevant evidence	N/A
Generalisability	21	Discuss the generalisability (external validity) of the study results	17-18
**Other information**			
Funding	22	Give the source of funding and the role of the funders for the present study and, if applicable, for the original study on which the present article is based	28

*Give information separately for cases and controls in case-control studies and, if applicable, for exposed and unexposed groups in cohort and cross-sectional studies.

Note: An Explanation and Elaboration article discusses each checklist item and gives methodological background and published examples of transparent reporting. The STROBE checklist is best used in conjunction with this article (freely available on the Web sites of PLoS Medicine at http://www.plosmedicine.org/, Annals of Internal Medicine at http://www.annals.org/, and Epidemiology at http://www.epidem.com/). Information on the STROBE Initiative is available at www.strobe-statement.org.

For peer review only

BMJ Open

Longitudinal access and exposure to green-blue spaces and individual-level mental health and wellbeing: A study programme protocol for a longitudinal, population-wide record-linked natural experiment

Journal:	*BMJ Open*
Manuscript ID	bmjopen-2018-027289.R1
Article Type:	Protocol
Date Submitted by the Author:	25-Oct-2018
Complete List of Authors:	Mizen, Amy; Swansea University, Heath Data Research-UK, Wales and Northern Ireland<br>Song, Jiao; Swansea University, Heath Data Research-UK, Wales and Northern Ireland<br>Fry, Richard; Swansea University, Heath Data Research-UK, Wales and Northern Ireland; Swansea University, National Centre for Population Health & Wellbeing Research<br>Akbari, Ashley; Swansea University, Heath Data Research-UK, Wales and Northern Ireland<br>Berridge, Damon; Swansea University, Heath Data Research-UK, Wales and Northern Ireland<br>Johnson, Rhodri; Swansea University, Heath Data Research-UK, Wales and Northern Ireland<br>Rebecca Lovell, Rebecca; University of Exeter Medical School, European Centre for Environment and Human Health<br>Lyons, Ronan; Swansea University, Heath Data Research-UK, Wales and Northern Ireland<br>Nieuwenhuijsen, Mark; Instituto de Salud Global de Barcelona (ISGlobal)<br>Stratton, Gareth; Swansea University, Heath Data Research-UK, Wales and Northern Ireland<br>Wheeler, Benedict; University of Exeter Medical School, European Centre for Environment and Human Health<br>White, James; Cardiff University, DECIPHer, Centre for Trials Research<br>White, Mathew; University of Exeter Medical School, European Centre for Environment and Human Health<br>Rodgers, Sarah; University of Liverpool, Department of Public Health and Policy; Swansea University, Heath Data Research-UK, Wales and Northern Ireland
**Primary Subject Heading**:	Public health
Secondary Subject Heading:	Mental health, Health informatics, Epidemiology
Keywords:	Wellbeing, Environmental exposure, Geographic Information Systems, Routine linked data, Longitudinal, MENTAL HEALTH

SCHOLARONE™
Manuscripts

Longitudinal access and exposure to green-blue spaces and individual-level mental health and wellbeing: A study programme protocol for a longitudinal, population-wide record-linked natural experiment

Amy Mizen[1], Jiao Song[1], Richard Fry[1,2], Ashley Akbari[1], Damon Berridge[1], Rhodri Johnson[1], Rebecca Lovell[3], Ronan A Lyons[1], Mark Nieuwenhuijsen[4], Gareth Stratton[1], Benedict W. Wheeler[3], James White[5], Mathew White[3], Sarah Rodgers[1,6*]

1. Heath Data Research-UK, Wales and Northern Ireland, Swansea University, Singleton Park, Swansea, UK; 2. National Centre for Population Health & Wellbeing Research, Swansea University, Singleton Park, Swansea, UK; 3. European Centre for Environment and Human Health, University of Exeter Medical School, Knowledge Spa, Royal Cornwall Hospital, Truro, Cornwall, UK 4. Instituto de Salud Global de Barcelona.c/ Rosselló, 132, 5º 2ª (08036), Barcelona, Spain 5. DECIPHer, Centre for Trials Research, Cardiff University, 4th floor Neaudd Merinionydd, Heath, Cardiff, UK 6. Department of Public Health and Policy, University of Liverpool, Liverpool, UK

*Correspondence to: Professor Sarah E Rodgers, Department of Public Health and Policy, University of Liverpool, Liverpool, UK; Sarah.Rodgers@Liverpool.ac.uk

AM and JS contributed equally to this paper.

Keywords

Mental health; Wellbeing; Environmental exposure; Geographic Information Systems; Routine linked data; Longitudinal

Abstract

Introduction: Studies suggest that access and exposure to green-blue spaces (GBS) have beneficial impacts on mental health. However, the evidence base is limited with respect to longitudinal studies. The main aim of this longitudinal, population-wide, record-linked natural experiment, is to model the daily lived experience by linking GBS accessibility indices, residential GBS exposure and health data; to enable quantification of the impact of GBS on wellbeing and common mental health disorders, for a national population.

For peer review only - http://bmjopen.bmj.com/site/about/guidelines.xhtml

Methods and analysis: This research will estimate the impact of neighbourhood GBS access, GBS exposure and visits to GBS on the risk of common mental health conditions and the opportunity for promoting Subjective wellbeing (SWB); both key priorities for public health. We will use Geographical Information System (GIS) to create quarterly household GBS accessibility indices and GBS exposure using digital map and satellite data for 1.4 million homes in Wales, UK (2008-2018). We will link the GBS accessibility indices and GBS exposures to individual level mental health outcomes for 1.7 million people with General Practitioner (GP) data and data from the National Survey for Wales (n = ~12,000) on wellbeing in the Secure Anonymised Information Linkage (SAIL) databank. We will examine if these associations are modified by multiple socio-physical variables, migration and socioeconomic disadvantage. Subgroup analyses will examine associations by different types of GBS. This longitudinal study will be augmented by cross-sectional research using survey data on self-reported visits to GBS and SWB.

Ethics and dissemination:

All data will be anonymised and linked within the privacy protecting SAIL Databank. We will be using anonymised data and therefore we are exempt from National Research Ethics Committee (NREC). An Information Governance Review Panel (IGRP) application [Project ID: 0562] to link these data has been approved.

The research programme will be undertaken in close collaboration with public/patient involvement groups. A multi-strategy programme of dissemination is planned with the academic community, policy makers, practitioners and the public.

Strengths and limitations of this study

- *This retrospective controlled evaluation of a natural experiment includes every adult in Wales, UK between 2008-2018, minimising selection bias*
- *Generating a national longitudinal dataset of changes in GBS exposure and access to GBS for households will reduce ecological fallacy*
- *Spatial and temporal accessibility data linked for individuals and their routinely captured health service use, together with potential confounders, will allow us to investigate the impact of GBS on wellbeing*
- *Detailed self-report data on wellbeing and GBS visit behaviour from cross-sectional national survey data will be linked to health service utilisation data*

For peer review only - http://bmjopen.bmj.com/site/about/guidelines.xhtml

- *Despite including a large number of potentially confounding variables, this non-randomised study using routinely recorded data may omit some unknown confounders, thereby introducing a moderate level of bias due to confounding*

For peer review only

For peer review only - http://bmjopen.bmj.com/site/about/guidelines.xhtml

Introduction

Globally, 686 million people suffer with common mental health disorders (CMD) such as depression or anxiety [1]. In the UK, CMD are experienced by around 1 in 4 of the population, and mental ill-health costs the economy over £100bn pa in health, social care and quality of life-loss costs [2,3]. Subjective wellbeing (SWB) is also related to mental and physical health outcomes, including life expectancy [4,5], and is a key marker of quality of life [6]. With increasing impacts on wider societal costs, CMD and promoting SWB are growing in importance. Access to natural environments – considered here as 'green-blue spaces' (GBS) – such as parks and beaches – may provide opportunities to support and promote good public mental health and wellbeing [7,8]. The evidence base on the impacts of GBS, on mental health and wellbeing is growing rapidly. Current research suggests that the benefits may differ by population group, context and health outcome [9,10].

Recent systematic reviews, of predominantly cross-sectional studies, indicate positive relationships between mental health and SWB outcomes with living near GBS [11,12]. Access to GBS is positively associated with GBS use, and using GBS may improve health outcomes through a number of mechanisms. For example, increased physical activity [13,14], psychological restoration [15,16], noise mitigation [17], heat and humidity regulation [18,19], increased social interaction and cohesion [20–22]. Contrary to these findings, a study in New Zealand found no evidence that GBS influenced cardiovascular disease mortality [23] and suggested that GBS and health relationships may vary according to country or environmental contexts.

Many studies have found that access to GBS can vary across socio-economic status (SES) areas; with more deprived areas tending to have poorer access to GBS [24,25]. Differences in the distribution of GBS across SES may influence and contribute to SES-related health inequalities [26]. Furthermore, individuals from higher SES groups are more likely to select living in greener neighbourhoods [27]. Previous studies lack censoring for births, deaths and migration, which may have led to health selection effects which can create/over/underestimate the strength of association. There are also a lack of large-scale population level studies which have systematically and explicitly examined associations within and between sub-groups other than SES [10] (e.g. by gender [28–30], age, education [22,31]). Furthermore, ethnic minority groups are underrepresented in studies

For peer review only - http://bmjopen.bmj.com/site/about/guidelines.xhtml

due to selective non-response [32]. Studies that have focussed on differences between ethnic minorities found positive effects of GBS on wellbeing differed by ethnicity [33] and ethnic minorities had poorer access to GBS [34].

Study methods used to define access to GBS differ; some studies have calculated small area-level metrics and others have used distances. There is no accepted method to define GBS or for measuring different types of GBS (e.g. according to general landcover categories, ecosystems or landscape classifications) [35–41]. However, it is difficult to expect an authoritative voice on defining GBS access because how people engage with GBS varies by population subgroup and environmental context. Therefore, how GBS is defined will vary by study design and the focus of the research. We propose to take an approach that will consider land use but also access points, rights of way and amenities such as benches. There are also differences in study aims because of differing perspectives; such as health research, environmental research and policy. Few studies have also considered the issue of access to blue spaces [42,43] which has also contributed to diversity in GBS accessibility measures. Although there is now a significant body of research, there are few longitudinal studies on mental health outcomes in response to changes in GBS access or with assessments of actual visits to GBS [44–46].

We define *GBS accessibility* indices as metrics that represent how people may access GBS (Figure 1). *GBS exposure* is the ambient environment that people experience because of where they live (Figure 1). By including both of these measures in our study, we will be able to build a more holistic view of how GBS impacts CMD and wellbeing.

For peer review only

Aim

Our primary aim is to quantify the impact of changes in access to GBS and ambient GBS exposure on CMD and SWB, for a national population. This will be fulfilled through a number of objectives within two work packages. Work package 1 (WP1) will use routinely collected health and demographic data (data collected for purposes other than research [47]) stored in an anonymised databank to examine the risk of CMD using longitudinal changes in access to neighbourhood GBS. Work package 2 (WP2) will link routine data in the anonymised databank with in-depth survey responses from the data linked National

For peer review only - http://bmjopen.bmj.com/site/about/guidelines.xhtml

Survey for Wales (NSW) to evaluate a representative sample of Welsh residents' self-reported wellbeing and GBS use.

Objectives

WP1

1. Create a longitudinal dataset of GBS in Wales using UK Ordnance Survey, Local Authority and remotely sensed satellite data;
2. Create longitudinal, residential GBS accessibility indices for all homes in Wales using the longitudinal GBS dataset in a GIS.
3. Create an 11-year dynamic cohort of individual-level longitudinal residential GBS accessibility indices to answer the research question(s): "*Do people with different GBS access through time have different associated risks of having a CMD?"*" and "*Is the association between changes in access to GBS and CMD modified by multiple socio-physical variables, migration and socioeconomic disadvantage?*"

WP2

1. Create data linkages between survey and routinely collected data within the Secure Anonymised Information Linkage databank (SAIL) for household-level GBS accessibility indices, and individual-level health and demographic data
2. Compliment residential GBS accessibility indices by including GBS usage from the data-linked National Survey for Wales to model interactions to answer research question: "*Is the association between GBS residential access, modified by GBS use and multiple socio-physical modifiers, on wellbeing, and common mental health disorders (CMD)*?"

Methods and analysis

Design

The GBS project is a retrospective and controlled, population-wide study. We will evaluate the association between changes in access to GBS, on the risk of CMD (WP1) and SWB (WP2). This could be either at environment level (e.g. a change in the GBS itself) or person level (e.g. moving home, better access to transport). WP1 will use longitudinal data to examine variability in time and space of access to GBS and examine whether this could

For peer review only - http://bmjopen.bmj.com/site/about/guidelines.xhtml

be due to planning and environmental policies. WP2 will use cross-sectional data to investigate whether visits to GBS improve SWB.

Participants

Our study population are made up of several different groups who are aged 16 years and older living in Wales, UK. WP1 includes the total adult population registered with a general practitioner (GP), providing GP records to SAIL. This is 1.7 million adults in Wales (see Figure 2). Dates for each update of the address record in a population register provide durations of residency for each successive address. WP2 includes a representative sample of the adult population in Wales based on the NSW for two years (cross-sectional samples in 2016/17 and 2017/18). The NSW has an annual sample of approximately 12,000 responders and GBS visit questions are asked of 50% of that sample. This provides a total cross-sectional sample of 12,000 (over the two years) for this part of the study.

Research Environment

Environment data will be collected, stored and processed to generate household level GBS accessibility indices. This work will be undertaken in a secure GIS platform which is hosted by UKSeRP [48]. High resolution map data from the Ordnance Survey (OS) is already stored in the GIS, including point data for all residences (Address Base Premium (OS ABP)) and road network data [49,50].

In parallel with, but separate from the GIS, is the Secure Anonymised Information Linkage (SAIL) Databank [51–53]. Data linkage and statistical analysis for this study will be conducted within the SAIL Databank. The SAIL Databank contains longitudinal health, social and education data on more than 5 million people which includes the active population of Wales, UK (3.1 million people) at any one time. The databank includes over 15 billion anonymised records and was designed to overcome data sharing issues.

A strength of the SAIL platform is the method for anonymising all individuals and households in Wales. Data are anonymised through the split-file process [51,52]. Whereby the dataset are separated in to identifiable (e.g. address) and non-identifiable (GBS access and exposure) components. The identifiable component is transported to our Trusted Third

For peer review only - http://bmjopen.bmj.com/site/about/guidelines.xhtml

Party (TTP), NHS Wales Informatics Service (NWIS) [54]. The non-identifiable component is sent securely to the SAIL databank. The TTP anonymise and encrypt the identifiable data and each individual record is assigned a unique linking field. An Anonymous Linking Field (ALF) is assigned to individual level data and a Residential Anonymous linking Field (RALF) is assigned to a place of residence [54]. The anonymised elements of the dataset are then sent to the SAIL databank to be loaded. These elements are then recombined with the non-identifiable (GBS access and exposure) component of the dataset, which makes them ready for linkage with other datasets in the SAIL databank (see Figure 3).

Data Sources

Environment Datasets

We will create a longitudinal, national dataset of GBS for Wales (2008-2018) using temporally and spatially referenced satellite imagery [55]. We will also develop a typology of GBS and collate data from multiple sources to augment the all-Wales OS data we hold in our GIS database (OS Mastermap) [49,50,56]. Using satellite imagery, we will extract temporal trends of 'greenness' (using measures such as the Normalised Difference Vegetation Index (NDVI), which has been used in previous studies e.g. [57]) for every household in Wales and we will also explore the integration of new GBS and land use data as they become available and are published (e.g. OS Greenspace data [58]) and local authority audits of GBS.

GBS Accessibility and Exposure Dataset

There is no clear consensus in the existing literature on the most appropriate measures of GBS access and/or exposure [42]. Using the aforementioned Environment datasets, this project will collate a set of measures using criteria including longitudinal consistency, spatial resolution and evidence for associations with mental health and SWB outcomes. We will create residential level exposure measures by modelling the ambient home environment i.e. the GBS immediately surrounding their home and what they see out of their window. This metric will include measures such as greenness (NDVI), urban trees and garden size. The access measures will represent people's potential to visit GBS in their locality. We will adapt a previous methodology that modelled 'change in alcohol outlet

For peer review only - http://bmjopen.bmj.com/site/about/guidelines.xhtml

density and alcohol-related harm to population health' (CHALICE) [59]. The distance decay for this accessibility model will be updated to emulate how people engage with GBS as opposed to alcohol outlets. People will behave differently in accessing GBS, compared with how they may access alcohol outlets. For example, people may be prepared to walk for a longer time and/or distance to access a GBS compared with alcohol outlets [60]. Our modelling will consider differential associations for key subgroups (e.g. deprived populations) thereby minimising risks that recommendations could increase inequalities. We will create a number of accessibility and exposure measures, allowing planners to consider the configuration (size, function, quality) of the most beneficial mix of spaces. We will also measure, for each adult, change in GBS access and exposure for the study period. Again, we will build on the CHALICE study [61] that measured change in alcohol outlet density by calculating change in density between the current and previous quarter and also the change between each quarter and baseline.

The longitudinal, household level, GBS accessibility and exposure dataset will be imported in to the SAIL Databank and linked with health datasets that are held within the SAIL Databank. Household identifiers (Unique Property Reference Numbers (UPRNs)) will be will be used to distinguish each household in the GIS and attached to each GBS accessibility and exposure index. Household identifiers will be replaced with an anonymised linking field within SAIL [62] (see Figure 3).

Health Datasets

Welsh Longitudinal General Practice (WLGP)

The Welsh Longitudinal General Practice (WLGP) dataset contains individual-level health data including Read codes for all diagnoses, symptoms and treatments recorded by a general practitioner (GP) for each person [63] from 2008-2018.

For peer review only - http://bmjopen.bmj.com/site/about/guidelines.xhtml

For peer review only

Welsh Demographic Service (WDS)

The Welsh Demographic Service (WDS) dataset contains addresses for all individuals who register with a GP. Dates for each address record update are recorded, thereby providing durations of residency for multiple homes and the ability to link to local environment indices at each time point. This dataset holds demographic data including age and gender. These data will be used to create population sub-groups based on age, gender and location for each period. The WDS contains historical patient provided address information linked anonymously at the individual level (the ALF) which is the primary key variable for record-linkage [51,52].

National Survey for Wales (NSW)

The National Survey for Wales (NSW) is a cross-sectional, representative sample of adult participants across Wales. Participants answer questions on public services such as education, transport, leisure activities, and self-reported health and well-being. They also report information on visits to GBS, such as visit frequency and activities undertaken during visits.

Figure 3 shows how GBS accessibility indices will be generated and linked with routinely collected health and survey data held within the SAIL databank. Table 1 shows the data sources and variables that we plan to derive from the routinely collected health data, survey dataset and population register that are available in the SAIL Databank.

For peer review only

For peer review only - http://bmjopen.bmj.com/site/about/guidelines.xhtml

For peer review only

For peer review only - http://bmjopen.bmj.com/site/about/guidelines.xhtml

Table 1. Data sources for outcome and accessibility/exposure variables to be used in statistical analysis for years 2008-2018

Dataset name	Data Source	Derived Variables	Coverage	Work package
GBS Accessibility and Exposure dataset	Local Authorities, Ordnance Survey, Satellite data sources	Typology to be developed. The dataset will include GBS such as parks, woodland, sports facilities, trees in residential areas	Wales	WP1 & WP2
Welsh Longitudinal General Practice (WLGP) dataset (core SAIL dataset)	Primary care records recorded by GP	CMDs such as anxiety and depression (see outcome variable section for details)	70% of practices in Wales provide data to SAIL	WP1 & WP2
Welsh Demographic Service dataset (core SAIL dataset)	NHS Wales Informatics Service	Age, gender, week of birth, multiple move in and out of home dates	Total population of Wales who are registered with a General Practitioner, a free to use service in the UK.	WP1 & WP2
National Survey for Wales (NSW) (restricted SAIL dataset)	Welsh Government	Subjective wellbeing, self-reported visits to GBS	Cross-sectional, representative sample approximately 12,000 participants per year	WP2

For peer review only

Statistical analysis

Outcome variables

(1) WP1 & 2: Primary Outcome – Common Mental Health Disorder (CMD)

Change in counts of CMD treatments for adults identified as CMD cases within the corresponding time periods for the 70% of adults in Wales for whom we have GP data records in SAIL (1.7M adults). Prevalence algorithms [64,65], detect cases of CMD (anxiety and depression) from routinely collected GP data. We will use the algorithm that incorporates historical diagnosis currently treaded with current diagnosis whether treated or untreated to identify CMD cases. For each adult, and for the time periods (quarters) identified as a CMD case, treatments will be counted per day and aggregated into quarterly counts. Figure 4 shows a conceptual model of exposure variable (GBS access and exposure) and primary outcome variable (CMD treatments).

(2) WP2: Secondary Outcome – Subjective Wellbeing (SWB)

SWB is measured by the Warwick-Edinburgh Mental Wellbeing Scale (WEMWBS) in the NSW for two survey years (2016/17 and 2017/18) for a representative population [66]. The WEMWBS is comprehensive (incorporating elements of both subjective and psychological wellbeing), short enough to be used in population-level surveys, responsive to change and has been validated among community samples of adults in the UK [67]. WEMWBS scores are on continuous scale from 14 to 70.

(3) WP1: Secondary outcome – GP events

We have the total number of events recorded for each person in a WLGP dataset. We will calculate the number of GP events, converting to a binary daily activity or no activity aggregated to quarterly counts. This eliminates double counting (e.g. counting the return of large quantities of test results only once). These are purposefully all events rather than those related only to CMD because this will capture people who frequently visit their GP but for whom no CMD diagnosis has been made. This will allow us to explore social isolation and as indicator of undiagnosed CMD.

For peer review only - http://bmjopen.bmj.com/site/about/guidelines.xhtml

(4) WP2: Secondary outcome – ONS Subjective Wellbeing

We will have individual responses in NSW to the 4 standard SWB questions (life satisfaction, sense of worth of activities, happiness and anxiety) as used by Office for National Statistics and the Organisation for Economic Co-operation and Development [68].As well as being relatable to national representative survey data, these data have also been used in previous GBS visit research using the comparable Monitor of Engagement with the Natural Environment for England [45].

Analysis plan

We will provide descriptive statistics of GBS access and exposure and changes in GBS access and exposure by household, LSOA, deprivation quintiles using WIMD and population characteristics. National Survey for Wales data will have descriptive statistics of each of the survey questions we will use (number of visits, labour market status, etc.).

Across both work packages, we have outcomes of two distinct types: continuous (SWB) and counts (CMD treatments, and GP event days). The continuous outcome will be intended to be analysed using a linear model. Poisson models will be used to analyse the longitudinal count data.

Our data are hierarchical in nature: WP1: three level data: LSOA/ Individual / Time (Quarter); WP2: two level data: LSOA/Individual. Accordingly, we will adopt a multilevel modelling approach. We will generalise the standard linear and Poisson models to handle two or three levels of variation, as appropriate. The resulting two- and three-level models will allow us to estimate the extent to which variation at each level may be explained by confounding variables (see Table 2). Having controlled for confounders, we will proceed to add access and exposure variables to our models, thereby allowing us to test for the significance of the access and exposure variables in the presence of the confounders. Inclusion of these variables in the above models will permit us to estimate their direct effects on our outcomes. Having included the main effects of both confounders and access and exposure variables, we will proceed

For peer review only - http://bmjopen.bmj.com/site/about/guidelines.xhtml

to test for the significance of selected pairwise interactions of interest, as shown in Table 2.

Table 2. Analysis Summary (P: Primary; S: Secondary)

WP	Outcomes (Type)	Exposures	Confounders	Interactions of interest
**1**	P: CMD (count)	P: Access and exposure to GBS	LSOA level: Quintile of deprivation (WIMD). Category of urban/rural settlement type (ONS classification)	Change in access and exposure to GBS by deprivation
	S: GP event days (count)	S: Time of move(s);	Individual level (SAIL): Gender, Age, Comorbidities	Change in access and exposure to GBS by time of move(s)
**2**	P2: SWB (continuous)	P2: Access and exposure to GBS	LSOA level: as per WP1.	Exact number of visits by individual level deprivation
	P1: CMD (count)	S: Level of engagement in 150+ minutes of moderate or vigorous intensity activity per week	Individual level and NSW: Gender, Age, Comorbidities , Highest educational qualification, Marital status (incl. living with partner)<br><br>Exact number of visits made outdoors for recreation in last 4 weeks	Exact number of visits by residential GBS

For peer review only - http://bmjopen.bmj.com/site/about/guidelines.xhtml

Discussion

This study will be the first of its kind to link GIS-generated GBS accessibility and exposure measures with routinely collected, longitudinal health data and cross-sectional survey data for a whole population. We plan to create a longitudinal GBS dataset for Wales. Using multi-source data we will build a dataset that records local level changes in GBS for Wales, for 11 years. Longitudinal studies that have previously examined GBS exposure have used cross-sectional environment data to calculate GBS accessibility and exposure indices. This may be because it is a resource and time-intensive task to create a longitudinal GBS dataset, and longitudinal data is not always available. In addition, bringing together data from different sources, harmonising the data and deriving GBS indices from the data requires expertise and specialised skills.

Our study will work with stakeholders and policy makers to develop a GBS typology that can be used to provide evidence that can be translated to help policy and practice. Improved evidence on the impacts of GBS on mental health is required to inform decisions relating to planning, area regeneration and environmental management. Natural England, England's statutory body responsible for the natural environment, concluded that a knowledge of causal pathways and contributory mechanisms that link mental health and environmental exposure is required [69]. This study will produce the evidence needed to address gaps stated by the Welsh Government Environment Bill White Paper [70] and inform implementation of the Well-being of Future Generations (Wales) Act 2015 [71]. We have key policy makers signed up as stakeholders for this project and they will be involved in discussions for how we define GBS, how we measure access and exposure to GBS and in the reporting of our results so that this study can be useful in generating evidence-based policy.

Another aim of the study is to create novel GBS accessibility indices and that will model GBS exposure in way that reflects how people may access GBS. The way that studies measure access to GBS is methodologically diverse and there is no general consensus on what is the best measure to use. Our measures will be informed by theories and findings in the literature and to compliment this, we will invite members of the public to workshops and ask them to help direct our research through a series

For peer review only - http://bmjopen.bmj.com/site/about/guidelines.xhtml

of focussed questions. The workshop group will comprise members who are experienced at considering the value of environment from the Health and Environment Public Engagement (HEPE) Group (hosted by University of Exeter), who will join Wales-based members of the public from urban park groups, and those experienced in considering data linkage proposals [72].

The overarching aim of our longitudinal study is to identify causal mechanisms to determine whether a positive change in access and exposure to GBS lowers the risk of CMD.

Patient and Public Involvement

Patients were not involved in the development of the research questions. However, during the development of exposure measurement, we engaged with SAIL Databank consumer panel providing us with the public perspectives. We will also engage with members in Health and Environment Public Engagement (HEPE) Group (hosted by University of Exeter) who are experienced at considering the value of environment. All members mentioned above will be updated with project progress.

Ethics and dissemination

Ethical arrangements

This study is based on routinely collected administrative, environment, and survey data. All data will be anonymised into a secure databank and therefore there will be no mechanism for informing potential study participants of possible benefits and known risks. We have obtained informed consent to use the linked and anonymised NSW data within the SAIL Databank. All routinely collected anonymised data held in SAIL, are exempt from consent due to the anonymised nature of the databank (under section 251, NREC).

Research Governance

We have applied and been granted approval by the independent Information Governance Review Panel (IGRP) for permission to conduct this study (study number 0562). The IGRP contains independent members from NREC and BMA, as well as lay

For peer review only - http://bmjopen.bmj.com/site/about/guidelines.xhtml

members, and have previously given permission for similar projects (e.g. NIHR PHR CHALICE and NIHR PHR Carmarthenshire Housing). The review process has checked that the study we are is useful, not service evaluation, and will not break anonymisation standards.

Dissemination Policy

We will regularly report our progress to the Study Steering Committee (SSC). The SSC will comprise academic experts and stakeholders (NRW, Keep Wales Tidy, Sport Wales and Welsh Government). At the end of the study we will hold a workshop to report our findings to stakeholders and the public. We will disseminate our findings to patient, policy and academic networks (e.g. Health Data Research-UK, Administrative Data Research and National Institute for Health Research) and we will present results to the public with easily accessible media (e.g. using infographics) to maximise international engagement. We will present findings via seminars to key health professionals, including Public Health England and Public Health Wales, health service commissioners, Local Authorities and government planning officials to make recommendations for future policy decisions in this area and to those who have an interest in improving GBS, CMD and promoting SWB. We also plan to publish papers in internationally peer reviewed journals to disseminate the research to the wider academic community and add to the evidence base. We will share our results at national and internationally recognised conferences and promote our findings in academic circles.

Abbreviations

ABP	Address Base Premium
BMA	British Medical Association
CHALICE	Change in alcohol outlet density and alcohol-related harm to population health
CMD	Common Mental Health Disorders
GBS	Green-Blue Spaces
GIS	Geographic Information Systems
GP	General Practitioner
HEPE	Health and Environment Public Engagement
IGRP	Information Governance Review Panel
LSOA	Local Super Output Area
NDVI	Normalised Difference Vegetation Index

For peer review only - http://bmjopen.bmj.com/site/about/guidelines.xhtml

NREC	National Research Ethics Committee
NRW	Natural Resources Wales
NSW	National Survey for Wales
ONS	Office for National Statistics
OS	Ordnance Survey
SAIL	Secure Anonymised Information Linkage
SSC	Study Steering Committee
SWB	Subjective Wellbeing
TTP	Trusted Third Party
UKSeRP	United Kingdom Secure e-Research Platform
UPRN	Unique Property Reference Number
WDS	Welsh Demographic Service
WEMWBS	Warwick-Edinburgh Mental Wellbeing Scale
WIMD	Welsh Index of Multiple deprivation
WLGP	Welsh Longitudinal General Practice
WP1	Work Package 1
WP2	Work Package 2

For peer review only

For peer review only - http://bmjopen.bmj.com/site/about/guidelines.xhtml

References

1 World Health Organization. Depression and other common mental disorders: global health estimates. *World Heal Organ* 2017;:1–24. doi:CC BY-NC-SA 3.0 IGO

2 Davies S. Annual Report of the Chief Medical Officer 2013. Public Mental Health Priorities: Investing in the Evidence. London: : Department of Health 2013.

3 Centre For Mental Health | Economic and social costs of mental health problems. https://www.centreformentalhealth.org.uk/economic-and-social-costs (accessed 13 Jun 2018).

4 Diener E, Chan MY. Happy People Live Longer: Subjective Well-Being Contributes to Health and Longevity. *Appl Psychol Heal Well-Being* 2011;**3**:1–43. doi:10.1111/j.1758-0854.2010.01045.x

5 Steptoe A, Deaton A, Stone AA. Subjective wellbeing, health, and ageing. *Lancet* 2015;**385**:640–8. doi:10.1016/S0140-6736(13)61489-0

6 WHO. The World Health Organization Quality of Life assessment (WHOQOL): position paper from the World Health Organization. *Soc Sci Med* 1995;**41**:1403–9. doi:10.1016/0277-9536(95)00112-K

7 Hartig T, Mitchell R, de Vries S, *et al.* Nature and Health. *Annu Rev Public Health* 2014;**35**:207–28. doi:10.1146/annurev-publhealth-032013-182443

8 Marmot M. Fair society, healthy lives. 2010.

9 Markevych I, Schoierer J, Hartig T, *et al.* Exploring pathways linking greenspace to health: Theoretical and methodological guidance. *Environ Res* 2017;**158**:301–17. doi:10.1016/j.envres.2017.06.028

10 Dadvand P, de Nazelle A, Figueras F, *et al.* Green space, health inequality and pregnancy. *Environ Int* 2012;**40**:110–5. doi:10.1016/j.envint.2011.07.004

11 Bowler DE, Buyung-Ali LM, Knight TM, *et al.* A systematic review of evidence for the added benefits to health of exposure to natural environments. *BMC Public Health* 2010;**10**:456. doi:10.1186/1471-2458-10-456

12 James P, Banay RF, Hart JE, *et al.* A Review of the Health Benefits of Greenness. *Curr Epidemiol Reports* 2015;**2**:131–42. doi:10.1007/s40471-015-0043-7

13 Thompson Coon J, Boddy K, Stein K, *et al.* Does participating in physical

For peer review only - http://bmjopen.bmj.com/site/about/guidelines.xhtml

activity in outdoor natural environments have a greater effect on physical and mental wellbeing than physical activity indoors? A systematic review. *Environ Sci Technol* 2011;**45**:1761–72. doi:10.1021/es102947t

14 Coombes E, Jones AP, Hillsdon M. The relationship of physical activity and overweight to objectively measured green space accessibility and use. *Soc Sci Med* 2010;**70**:816–22. doi:10.1016/j.socscimed.2009.11.020

15 White MP, Alcock I, Wheeler BW, *et al.* Would You Be Happier Living in a Greener Urban Area? A Fixed-Effects Analysis of Panel Data. *Psychol Sci* 2013;:0956797612464659. doi:10.1177/0956797612464659

16 White MP, Alcock I, Wheeler BW, *et al.* Coastal proximity, health and well-being: Results from a longitudinal panel survey. *Heal Place* 2013;**23**:97–103. doi:10.1016/j.healthplace.2013.05.006

17 Hystad P, Davies HW, Frank L, *et al.* Residential Greenness and Birth Outcomes: Evaluating the Influence of Spatially Correlated Built-Environment Factors. *Environ Health Perspect* 2014;**122**:1095–102. doi:10.1289/ehp.1308049

18 Dadvand P, Sunyer J, Basagaña X, *et al.* Surrounding greenness and pregnancy outcomes in four Spanish birth cohorts. *Environ Health Perspect* 2012;**120**:1481–7. doi:10.1289/ehp.1205244

19 Kingsley SL, Eliot MN, Whitsel EA, *et al.* Maternal residential proximity to major roadways, birth weight, and placental DNA methylation. *Environ Int* 2016;**92–93**:43–9. doi:10.1016/j.envint.2016.03.020

20 De Vries S, van Dillen SME, Groenewegen PP, *et al.* Streetscape greenery and health: Stress, social cohesion and physical activity as mediators. *Soc Sci Med* 2013;**94**:26–33. doi:10.1016/j.socscimed.2013.06.030

21 van den Berg MM, van Poppel M, van Kamp I, *et al.* Do Physical Activity, Social Cohesion, and Loneliness Mediate the Association Between Time Spent Visiting Green Space and Mental Health? *Environ Behav* 2017;:001391651773856. doi:10.1177/0013916517738563

22 Maas J, van Dillen SME, Verheij RA, *et al.* Social contacts as a possible mechanism behind the relation between green space and health. *Heal Place* 2009;**15**:586–95. doi:10.1016/j.healthplace.2008.09.006

23 Richardson E, Pearce J, Mitchell R, *et al.* The association between green space and cause-specific mortality in urban New Zealand: an ecological

For peer review only

For peer review only - http://bmjopen.bmj.com/site/about/guidelines.xhtml

analysis of green space utility. *BMC Public Health* 2010;**10**:240. doi:10.1186/1471-2458-10-240

24 Mitchell R, Astell-Burt T, Richardson EA. A comparison of green space indicators for epidemiological research. *J Epidemiol Community Health* 2011;**65**:853–8. doi:10.1136/jech.2010.119172

25 Mitchell R, Popham F. Effect of exposure to natural environment on health inequalities: an observational population study. *Lancet* 2008;**372**:1655–60. doi:10.1016/S0140-6736(08)61689-X

26 Sugiyama T, Villanueva K, Knuiman M, *et al.* Can neighborhood green space mitigate health inequalities? A study of socio-economic status and mental health. *Heal Place* 2016;**38**:16–21. doi:10.1016/j.healthplace.2016.01.002

27 Amoly E, Dadvand P, Forns J, *et al.* Green and blue spaces and behavioral development in barcelona schoolchildren: The BREATHE project. *Environ Health Perspect* 2015;**122**:1351–8. doi:10.1289/ehp.1408215

28 Astell-Burt T, Mitchell R, Hartig T. The association between green space and mental health varies across the lifecourse. a longitudinal study. *J Epidemiol Community Health* 2014;**68**:578–83. doi:10.1136/jech-2013-203767

29 Mitchell R, Popham F. Greenspace, urbanity and health: Relationships in England. *J Epidemiol Community Health* 2007;**61**:681–3. doi:10.1136/jech.2006.053553

30 Reklaitiene R, Grazuleviciene R, Dedele A, *et al.* The relationship of green space, depressive symptoms and perceived general health in urban population. *Scand J Public Health* 2014;**42**:669–76. doi:10.1177/1403494814544494

31 de Vries S, Verheij RA, Groenewegen PP, *et al.* Natural environments - Healthy environments? An exploratory analysis of the relationship between greenspace and health. *Environ Plan A* 2003;**35**:1717–31. doi:10.1068/a35111

32 van den Berg M, Wendel-Vos W, van Poppel M, *et al.* Health benefits of green spaces in the living environment: A systematic review of epidemiological studies. *Urban For Urban Green* 2015;**14**:806–16. doi:10.1016/j.ufug.2015.07.008

33 McEachan RRC, Yang TC, Roberts H, *et al.* Availability, use of, and satisfaction with green space, and children's mental wellbeing at age 4 years in a multicultural, deprived, urban area: results from the Born in Bradford

For peer review only - http://bmjopen.bmj.com/site/about/guidelines.xhtml

cohort study. *Lancet Planet Heal* 2018;**2**:e244–54. doi:10.1016/S2542-5196(18)30119-0

34 Dai D. Racial/ethnic and socioeconomic disparities in urban green space accessibility: Where to intervene? *Landsc Urban Plan* 2011;**102**:234–44. doi:10.1016/j.landurbplan.2011.05.002

35 Coolen H, Meesters J. Private and public green spaces: Meaningful but different settings. *J Hous Built Environ* 2012;**27**:49–67. doi:10.1007/s10901-011-9246-5

36 Taylor L, Hochuli DF. Defining greenspace: Multiple uses across multiple disciplines. *Landsc Urban Plan* 2017;**158**:25–38. doi:10.1016/j.landurbplan.2016.09.024

37 Wheeler BW, Lovell R, Higgins SL, *et al.* Beyond greenspace: an ecological study of population general health and indicators of natural environment type and quality. *Int J Health Geogr* 2015;**14**:17. doi:10.1186/s12942-015-0009-5

38 Alcock I, White MP, Lovell R, *et al.* What accounts for 'England's green and pleasant land'? A panel data analysis of mental health and land cover types in rural England. *Landsc Urban Plan* 2015;**142**:38–46. doi:10.1016/j.landurbplan.2015.05.008

39 MacKerron G, Mourato S. Happiness is greater in natural environments. *Glob Environ Chang* 2013;**23**:992–1000. doi:10.1016/j.gloenvcha.2013.03.010

40 van Dillen SME, de Vries S, Groenewegen PP, *et al.* Greenspace in urban neighbourhoods and residents' health: Adding quality to quantity. *J Epidemiol Community Health* 2012;**66**:1–5. doi:10.1136/jech.2009.104695

41 Annerstedt M, Östergren P-O, Björk J, *et al.* Green qualities in the neighbourhood and mental health – results from a longitudinal cohort study in Southern Sweden. *BMC Public Health* 2012;**12**:337. doi:10.1186/1471-2458-12-337

42 Ekkel ED, de Vries S. Nearby green space and human health: Evaluating accessibility metrics. *Landsc Urban Plan* 2017;**157**:214–20. doi:10.1016/j.landurbplan.2016.06.008

43 Grellier J, White MP, Albin M, *et al.* BlueHealth: A study programme protocol for mapping and quantifying the potential benefits to public health and well-being from Europe's blue spaces. *BMJ Open* 2017;**7**:1–10. doi:10.1136/bmjopen-2017-016188

For peer review only - http://bmjopen.bmj.com/site/about/guidelines.xhtml

44 Gascon M, Triguero-Mas M, Martínez D, *et al.* Mental Health Benefits of Long-Term Exposure to Residential Green and Blue Spaces: A Systematic Review. *Int J Environ Res Public Health* 2015;**12**:4354–79. doi:10.3390/ijerph120404354

45 White MP, Pahl S, Wheeler BW, *et al.* Natural environments and subjective wellbeing: Different types of exposure are associated with different aspects of wellbeing. *Heal Place* 2017;**45**:77–84. doi:10.1016/j.healthplace.2017.03.008

46 White MP, Pahl S, Ashbullby K, *et al.* Feelings of restoration from recent nature visits. *J Environ Psychol* 2013;**35**:40–51. doi:10.1016/j.jenvp.2013.04.002

47 Nicholls SG, Langan SM, Benchimol EI. Routinely collected data: The importance of high-quality diagnostic coding to research. *Cmaj* 2017;**189**:E1054–5. doi:10.1503/cmaj.170807

48 Health Informatics Group SUMS. UK Secure eResearch Platform UKSeRP Data Sharing : Why Should We Share Data? Swansea: 2017. https://saildatabank.com/wp-content/uploads/UKSeRP_Brochure_v1.5.pdf

49 AddressBase Premium | Business and government | Ordnance Survey. http://www.ordnancesurvey.co.uk/business-and-government/products/addressbase-premium.html (accessed 10 Mar 2014).

50 Ordnance Survey. OS(R) MasterMap(R) Integrated Transportation NetworkTM Layer technical specification. (c) Crown copyright 2011. 2012.http://www.ordnancesurvey.co.uk/oswebsite/support/products/os-mastermap/itn-layer-technical-specification/index.html%5Cnhttp://www.ordnancesurvey.co.uk/oswebsite/docs/user-guides/points-of-interest-user-guide.pdf%5Cnhttp://www.ordnancesurvey.co.uk/oswebsi

51 Lyons R a, Jones KH, John G, *et al.* The SAIL databank: linking multiple health and social care datasets. *BMC Med Inform Decis Mak* 2009;**9**:3. doi:10.1186/1472-6947-9-3

52 Ford D V., Jones KH, Verplancke J-P, *et al.* The SAIL Databank: building a national architecture for e-health research and evaluation. *BMC Health Serv Res* 2009;**9**:157. doi:10.1186/1472-6963-9-157

53 Rodgers SE, Lyons RA, Dsilva R, *et al.* Residential Anonymous Linking Fields (RALFs): A novel information infrastructure to study the interaction between

For peer review only - http://bmjopen.bmj.com/site/about/guidelines.xhtml

the environment and individuals' health. *J Public Health (Bangkok)* 2009;**31**:582–8. doi:10.1093/pubmed/fdp041

54 SAIL Databank - The Secure Anonymised Information Linkage Databank. https://saildatabank.com/saildata/data-privacy-security/#protecting-identities (accessed 3 Oct 2017).

55 Gan M, Deng J, Zheng X, *et al.* Monitoring urban greenness dynamics using multiple endmember spectral mixture analysis. *PLoS One* 2014;**9**. doi:10.1371/journal.pone.0112202

56 Ordnance Survey. OS MasterMap Topography Layer. 2017.https://www.ordnancesurvey.co.uk/business-and-government/products/topography-layer.html (accessed 15 Nov 2017).

57 Beyer KMM, Kaltenbach A, Szabo A, *et al.* Exposure to neighborhood green space and mental health: Evidence from the survey of the health of wisconsin. *Int J Environ Res Public Health* 2014;**11**:3453–72. doi:10.3390/ijerph110303453

58 OS Greenspace | OS GetOutside.

59 Fry R, Orford S, Rodgers S. A best practice framework to measure spatial variation in alcohol availability. *Environ Plan B Plan Des* 2018;**0**:1–19. doi:10.1177/2399808318773761

60 Fone D, Morgan J, Fry R, *et al.* Change in alcohol outlet density and alcohol-related harm to population health (CHALICE): a comprehensive record-linked database study in Wales. *Chang alcohol outlet density alcohol-related harm to Popul Heal a Compr Rec database study Wales* 2016;**4**. doi:10.3310/phr04030

61 Fone D, Dunstan F, Lee S, *et al.* Change in alcohol outlet density and alcohol-related harm to population health (CHALICE): A comprehensive record-linked database study in Wales. 2015.http://www.nets.nihr.ac.uk/projects/phr/09300702 (accessed 24 Jun 2015).

62 Rodgers SE, Lyons RA, Dsilva R, *et al.* Residential Anonymous Linking Fields (RALFs): A Novel Information Infrastructure to Study the Interaction between the Environment and Individuals' Health. *J Public Health (Bangkok)* 2009;**10**:1–7. doi:10.1093/pubmed/fdp041

63 Read Codes - NHS Digital. https://digital.nhs.uk/article/1104/Read-Codes (accessed 19 Dec 2017).

For peer review only - http://bmjopen.bmj.com/site/about/guidelines.xhtml

64 John A, McGregor J, Fone D, *et al.* Case-finding for common mental disorders of anxiety and depression in primary care: an external validation of routinely collected data. *BMC Med Inform Decis Mak* 2016;**16**:35. doi:10.1186/s12911-016-0274-7

65 Cornish RP, John A, Boyd A, *et al.* Defining adolescent common mental disorders using electronic primary care data: a comparison with outcomes measured using the CIS-R. *BMJ Open* 2016;**6**:e013167. doi:10.1136/bmjopen-2016-013167

66 Aumeyr M, Brown Z, Doherty R, *et al.* National Survey for Wales 2016-17 Technical Report (Updated) National Survey for Wales 2016-17 Technical Report. Published Online First: 2017.http://doc.ukdataservice.ac.uk/doc/8301/mrdoc/pdf/8301_171018-national-survey-wales-2016-17-technical-report-en.pdf (accessed 19 Jun 2018).

67 Tennant R, Hiller L, Fishwick R, *et al.* The Warwick-Edinburgh Mental Well-being Scale (WEMWBS): development and UK validation. *Health Qual Life Outcomes* 2007;**5**:63. doi:10.1186/1477-7525-5-63

68 Personal Well-being user guidance - Office for National Statistics. https://www.ons.gov.uk/peoplepopulationandcommunity/wellbeing/methodologies/personalwellbeingsurveyuserguide (accessed 30 Aug 2018).

69 Natural England. Links between natural environments and mental health: evidence briefing - EIN018. 2016. publications.naturalengland.org.uk/file/468126390707814

70 Welsh Government. White Paper Towards the Sustainable Management of Wales ’ Natural Resources, Consultation on proposals for an Environmental Bill, WG19631. 2013.

71 Welsh Government | Well-being of Future Generations (Wales) Act 2015. http://gov.wales/topics/people-and-communities/people/future-generations-act/?lang=en (accessed 7 Nov 2017).

72 SAIL Databank - Consumer Panel. https://saildatabank.com/about-us/public-engagement/ (accessed 12 Jul 2018).

For peer review only - http://bmjopen.bmj.com/site/about/guidelines.xhtml

Contributors

AM and JS contributed equally to writing this paper. This paper was based on a grant proposal developed by SR, RF, AA, DB, RJ, BW, RL, RL, MN, GS, BW, JW and MW. This paper was written based on a draft manuscript written by RF based on the grant proposal. Specifically, the Introduction, and Discussion was written by AM. JS wrote the Analysis plan and developed the conceptual model. The Methods section was written by AM and JS. All the co-authors made substantial contributions to the writing of the Methods section and editing of the whole paper. All authors then read the final version and approved it for submission and publication.

Funding

Funding acknowledgement

This project was funded by the NIHR PHR (project number 16/07/07)

Department of Health and Social Care disclaimer

The views expressed are those of the author(s) and not necessarily those of the NHS, the NIHR or the Department of Health and Social Care.

This work was supported by Health Data Research UK (NIWA1), which is funded by the UK Medical Research Council, Engineering and Physical Sciences Research Council, Economic and Social Research Council, National Institute for Health Research (England), Chief Scientist Office of the Scottish Government Health and Social Care Directorates, Health and Social Care Research and Development Division (Welsh Government), Public Health Agency (Northern Ireland), British Heart Foundation and Wellcome. This work was supported by the National Centre for Population Health and Well-Being Research (NXPHWR).

Acknowledgement

This study makes use of anonymised data held in the Secure Anonymised Information Linkage (SAIL) Databank. We would like to acknowledge all the data providers who make anonymised data available for research. We would like to acknowledge SAIL Databank consumer panel for providing us with the public perspectives.

For peer review only - http://bmjopen.bmj.com/site/about/guidelines.xhtml

For peer review only

Competing interests

None

Figure 1. Residential GBS access and exposure for each home 1) Household exposure defined as greenness immediately surrounding home/what can be seen out of the window. 2) Network buffer defines household access boundary (e.g. 10-minute walk). 3) GBS A and B included in access estimates including access points, rights of way and facilities such as benches (GBS C excluded). 3) Access measures include GBS quality, size, function and weighted network distances (d_i) to access points. 4) Longitudinal exposure estimates created through quarterly repeat analysis.

Figure 2. Flow diagram of proposed number (1.7 million adults) we currently have before exposure group allocation.

Figure 3: Diagram of proposed GIS data preparation and data linkage in SAIL Databank. GBS accessibility and exposure indices created within a secure GIS platform will be linked at household level with demographics from Welsh Demographic Service (WDS) and National Survey for Wales (NSW). Common mental health conditions from GP records will be linked at individual level with the NSW and WDS. Linkage will be undertaken by our Trusted Third Party (NHS Wales Informatics Service – NWIS).

Figure 4: Conceptual model of primary exposure variable and primary outcome variable.

For peer review only

For peer review only - http://bmjopen.bmj.com/site/about/guidelines.xhtml

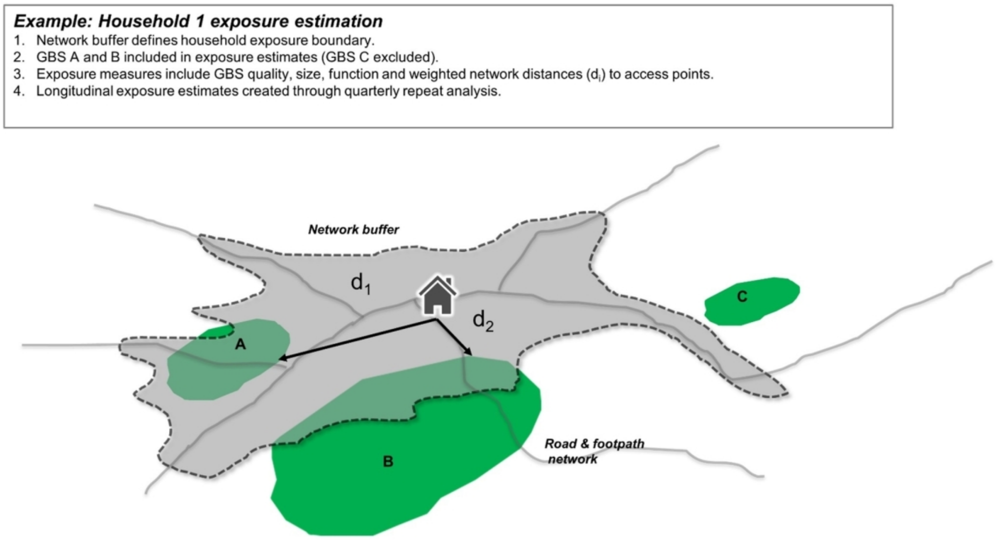

Figure 1. Residential GBS access and exposure for each home 1) Household exposure defined as greenness immediately surrounding home/what can be seen out of the window. 2) Network buffer defines household access boundary (e.g. 10-minute walk). 3) GBS A and B included in access estimates including access points, rights of way and facilities such as benches (GBS C excluded). 3) Access measures include GBS quality, size, function and weighted network distances (di) to access points. 4) Longitudinal exposure estimates created through quarterly repeat analysis.

159x90mm (300 x 300 DPI)

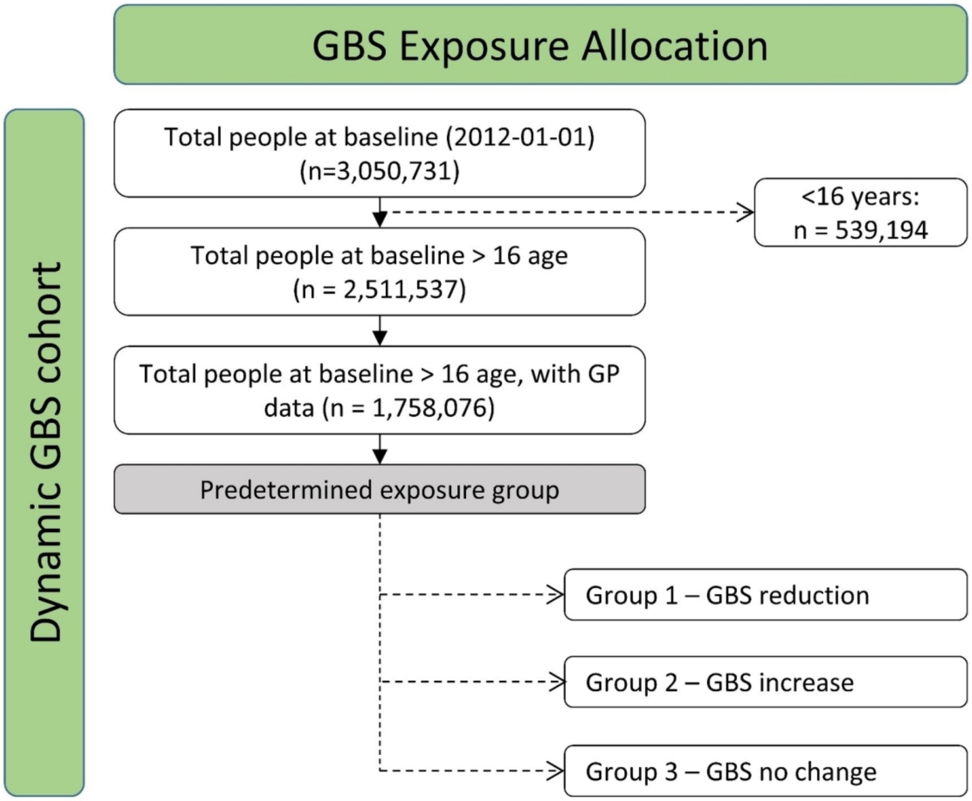

Figure 2. Flow diagram of proposed number (1.7 million adults) we currently have before exposure group allocation.

136x113mm (300 x 300 DPI)

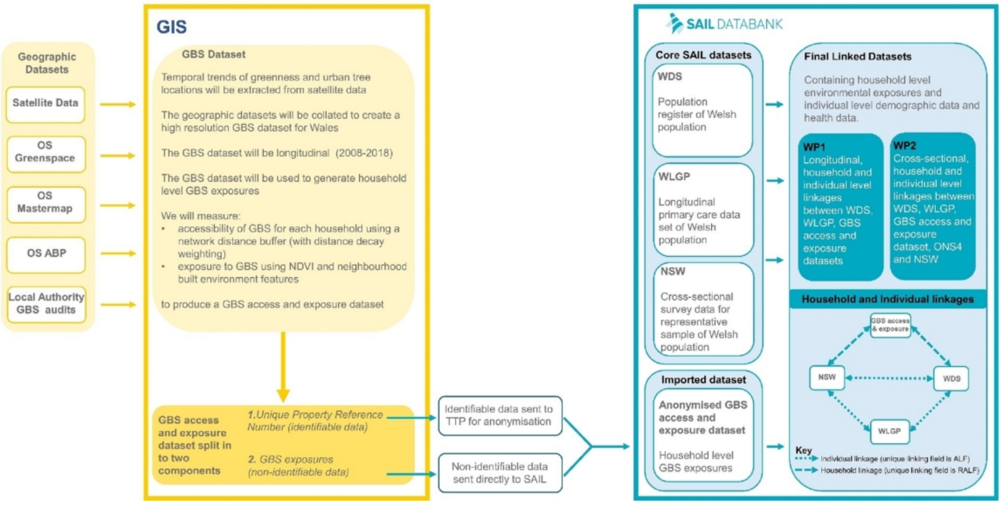

Figure 3: Diagram of proposed GIS data preparation and data linkage in SAIL Databank. GBS accessibility and exposure indices created within a secure GIS platform will be linked at household level with demographics from Welsh Demographic Service (WDS) and National Survey for Wales (NSW). Common mental health conditions from GP records will be linked at individual level with the NSW and WDS. Linkage will be undertaken by our Trusted Third Party (NHS Wales Informatics Service – NWIS).

206x104mm (300 x 300 DPI)

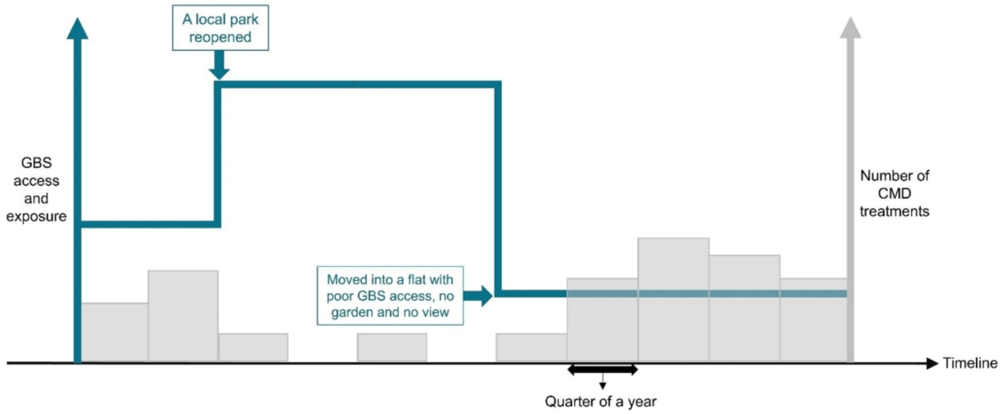

Figure 4: Conceptual model of primary exposure variable and primary outcome variable.

238x98mm (300 x 300 DPI)

STROBE Statement—checklist of items that should be included in reports of observational studies

	Item No.	Recommendation	Page No.
**Title and abstract**	1	(*a*) Indicate the study's design with a commonly used term in the title or the abstract	1
		(*b*) Provide in the abstract an informative and balanced summary of what was done and what was found	1-2
**Introduction**			
Background/rationale	2	Explain the scientific background and rationale for the investigation being reported	3-5
Objectives	3	State specific objectives, including any prespecified hypotheses	5-6
**Methods**			
Study design	4	Present key elements of study design early in the paper	6-12
Setting	5	Describe the setting, locations, and relevant dates, including periods of recruitment, exposure, follow-up, and data collection	6-12
Participants	6	(*a*) *Cohort study*—Give the eligibility criteria, and the sources and methods of selection of participants. Describe methods of follow-up<br>*Case-control study*—Give the eligibility criteria, and the sources and methods of case ascertainment and control selection. Give the rationale for the choice of cases and controls<br>*Cross-sectional study*—Give the eligibility criteria, and the sources and methods of selection of participants	6-12
		(*b*) *Cohort study*—For matched studies, give matching criteria and number of exposed and unexposed<br>*Case-control study*—For matched studies, give matching criteria and the number of controls per case	
Variables	7	Clearly define all outcomes, exposures, predictors, potential confounders, and effect modifiers. Give diagnostic criteria, if applicable	13-14
Data sources/ measurement	8*	For each variable of interest, give sources of data and details of methods of assessment (measurement). Describe comparability of assessment methods if there is more than one group	13-14
Bias	9	Describe any efforts to address potential sources of bias	2
Study size	10	Explain how the study size was arrived at	7

Continued on next page

For peer review only - http://bmjopen.bmj.com/site/about/guidelines.xhtml

Quantitative variables	11	Explain how quantitative variables were handled in the analyses. If applicable, describe which groupings were chosen and why	13-16
Statistical methods	12	(*a*) Describe all statistical methods, including those used to control for confounding	14-16
		(*b*) Describe any methods used to examine subgroups and interactions	14-16
		(*c*) Explain how missing data were addressed	N/A
		(*d*) *Cohort study*—If applicable, explain how loss to follow-up was addressed<br>*Case-control study*—If applicable, explain how matching of cases and controls was addressed<br>*Cross-sectional study*—If applicable, describe analytical methods taking account of sampling strategy	N/A
		(*e*) Describe any sensitivity analyses	N/A
**Results**			
Participants	13*	(a) Report numbers of individuals at each stage of study—eg numbers potentially eligible, examined for eligibility, confirmed eligible, included in the study, completing follow-up, and analysed	N/A
		(b) Give reasons for non-participation at each stage	N/A
		(c) Consider use of a flow diagram	N/A
Descriptive data	14*	(a) Give characteristics of study participants (eg demographic, clinical, social) and information on exposures and potential confounders	N/A
		(b) Indicate number of participants with missing data for each variable of interest	N/A
		(c) *Cohort study*—Summarise follow-up time (eg, average and total amount)	N/A
Outcome data	15*	*Cohort study*—Report numbers of outcome events or summary measures over time	N/A
		Case-control study—Report numbers in each exposure category, or summary measures of exposure	N/A
		Cross-sectional study—Report numbers of outcome events or summary measures	
Main results	16	(*a*) Give unadjusted estimates and, if applicable, confounder-adjusted estimates and their precision (eg, 95% confidence interval). Make clear which confounders were adjusted for and why they were included	N/A
		(*b*) Report category boundaries when continuous variables were categorized	N/A
		(*c*) If relevant, consider translating estimates of relative risk into absolute risk for a meaningful time period	N/A

Continued on next page

For peer review only

Other analyses	17	Report other analyses done—eg analyses of subgroups and interactions, and sensitivity analyses	N/A
**Discussion**			
Key results	18	Summarise key results with reference to study objectives	N/A
Limitations	19	Discuss limitations of the study, taking into account sources of potential bias or imprecision. Discuss both direction and magnitude of any potential bias	17-18
Interpretation	20	Give a cautious overall interpretation of results considering objectives, limitations, multiplicity of analyses, results from similar studies, and other relevant evidence	N/A
Generalisability	21	Discuss the generalisability (external validity) of the study results	17-18
**Other information**			
Funding	22	Give the source of funding and the role of the funders for the present study and, if applicable, for the original study on which the present article is based	28

*Give information separately for cases and controls in case-control studies and, if applicable, for exposed and unexposed groups in cohort and cross-sectional studies.

Note: An Explanation and Elaboration article discusses each checklist item and gives methodological background and published examples of transparent reporting. The STROBE checklist is best used in conjunction with this article (freely available on the Web sites of PLoS Medicine at http://www.plosmedicine.org/, Annals of Internal Medicine at http://www.annals.org/, and Epidemiology at http://www.epidem.com/). Information on the STROBE Initiative is available at www.strobe-statement.org.

For peer review only

For peer review only - http://bmjopen.bmj.com/site/about/guidelines.xhtml
