# Supplementary material for: Longitudinal access and exposure to green-blue spaces and individual-level mental health and well-being: protocol for a longitudinal, population-wide record-linked natural experiment
Source: BMJ Open. 2019 Apr 20;9(4):e027289. doi: 10.1136/bmjopen-2018-027289 (PMC6528002; doi:10.1136/bmjopen-2018-027289)
Supplement: Reviewer comments [file bmjopen-2018-027289.reviewer_comments.pdf]

PEER REVIEW HISTORY

BMJ Open publishes all reviews undertaken for accepted manuscripts. Reviewers are asked to complete a checklist review form (**http://bmjopen.bmj.com/site/about/resources/checklist.pdf**) and are provided with free text boxes to elaborate on their assessment. These free text comments are reproduced below.

ARTICLE DETAILS

**TITLE (PROVISIONAL)**	Longitudinal access and exposure to green-blue spaces and individual-level mental health and wellbeing: A study programme protocol for a longitudinal, population-wide record-linked natural experiment
**AUTHORS**	Mizen, Amy; Song, Jiao; Fry, Richard; Akbari, Ashley; Berridge, Damon; Johnson, Rhodri; Rebecca Lovell, Rebecca; Lyons, Ronan; Nieuwenhuijsen, Mark; Stratton, Gareth; Wheeler, Benedict; White, James; White, Mathew; Rodgers, Sarah

This article was not externally reviewed at BMJ Open. Protocols that have been independently assessed prior to submission to BMJ Open are usually fast-tracked to publication on the grounds that further substantial changes will not be possible. This independent assessment will usually be external, independent review for both a substantial grant award from a non-commercial or government funder and ethics approval.
